# Recent Advances in Histidine Kinase-Targeted Antimicrobial Agents

**DOI:** 10.3389/fchem.2022.866392

**Published:** 2022-07-04

**Authors:** Hongtong Chen, Chengqi Yu, Han Wu, Guoqing Li, Congran Li, Wei Hong, Xinyi Yang, Hao Wang, Xuefu You

**Affiliations:** ^1^ Laboratory of Pharmacology/Beijing Key Laboratory of Antimicrobial Agents, Institute of Medicinal Biotechnology, Chinese Academy of Medical Sciences and Peking Union Medical College, Beijing, China; ^2^ School of Basic Medical Science, Capital Medical University, Beijing, China; ^3^ School of Pharmacy, Minzu University of China, Beijing, China; ^4^ Key Laboratory of Ethnomedicine (Minzu University of China), Ministry of Education, Beijing, China; ^5^ Beijing Institute of Collaborative Innovation, Beijing, China; ^6^ Institute of National Security, Minzu University of China, Beijing, China

**Keywords:** histidine kinases, histidine kinase inhibitors, antibacterial agents, antivirulence agents, two-component system

## Abstract

The prevalence of antimicrobial-resistant pathogens significantly limited the number of effective antibiotics available clinically, which urgently requires new drug targets to screen, design, and develop novel antibacterial drugs. Two-component system (TCS), which is comprised of a histidine kinase (HK) and a response regulator (RR), is a common mechanism whereby bacteria can sense a range of stimuli and make an appropriate adaptive response. HKs as the sensor part of the bacterial TCS can regulate various processes such as growth, vitality, antibiotic resistance, and virulence, and have been considered as a promising target for antibacterial drugs. In the current review, we highlighted the structural basis and functional importance of bacterial TCS especially HKs as a target in the discovery of new antimicrobials, and summarize the latest research progress of small-molecule HK-inhibitors as potential novel antimicrobial drugs reported in the past decade.

## 1 Background

### 1.1 The Serious Problem of Antimicrobial Resistance

Antibiotics are one of the greatest medical achievements of mankind in the 20th century and have revolutionised the treatment of patients with infections or infectious diseases worldwide. The discovery of penicillin in 1928 initiated the golden age of antibiotic discovery from natural products, and a peak occurred in the mid-1950s (Hutchings et al., 2019). Antibiotics are the main treatment for the elimination of bacterial infections and have been widely used in numerous other settings beyond healthcare (Prescott, 2014). However, with the widespread, frequent, and inappropriate use of antibiotics, bacteria resistance to commonly employed antimicrobials has become increasingly prevalent in both healthcare and community settings, leading to higher medical costs, prolonged hospital stays, and increased mortality rates. Unfortunately, the development of new classes of antimicrobial drugs gradually declined after the 1970s (Hutchings et al., 2019; Imchen et al., 2020). In the past two decades, only a few antibiotics with new mechanisms of action, such as oxazolidinones (i.e., linezolid) and lipopeptides (i.e., daptomycin), have been developed (Azzouz and Preuss, 2020; Patel and Saw, 2020). Moreover, the evolution of drug-resistant pathogens has significantly limited the number of clinically usable antibiotics, and antimicrobial resistance has been characterized as a global health threat, with 1.27 million deaths being directly attributable to antimicrobial resistance in 2019 alone (Antimicrobial Resistance Collaborators, 2022). Thus, the development of new antimicrobial agents with new structures and unique mechanisms of action is urgently needed for controlling the crisis of antimicrobial resistance (Barriere, 2015).

### 1.2 Structure and Fundamentals of Bacterial Two-Component Signal Transduction

**FIGURE 1:**
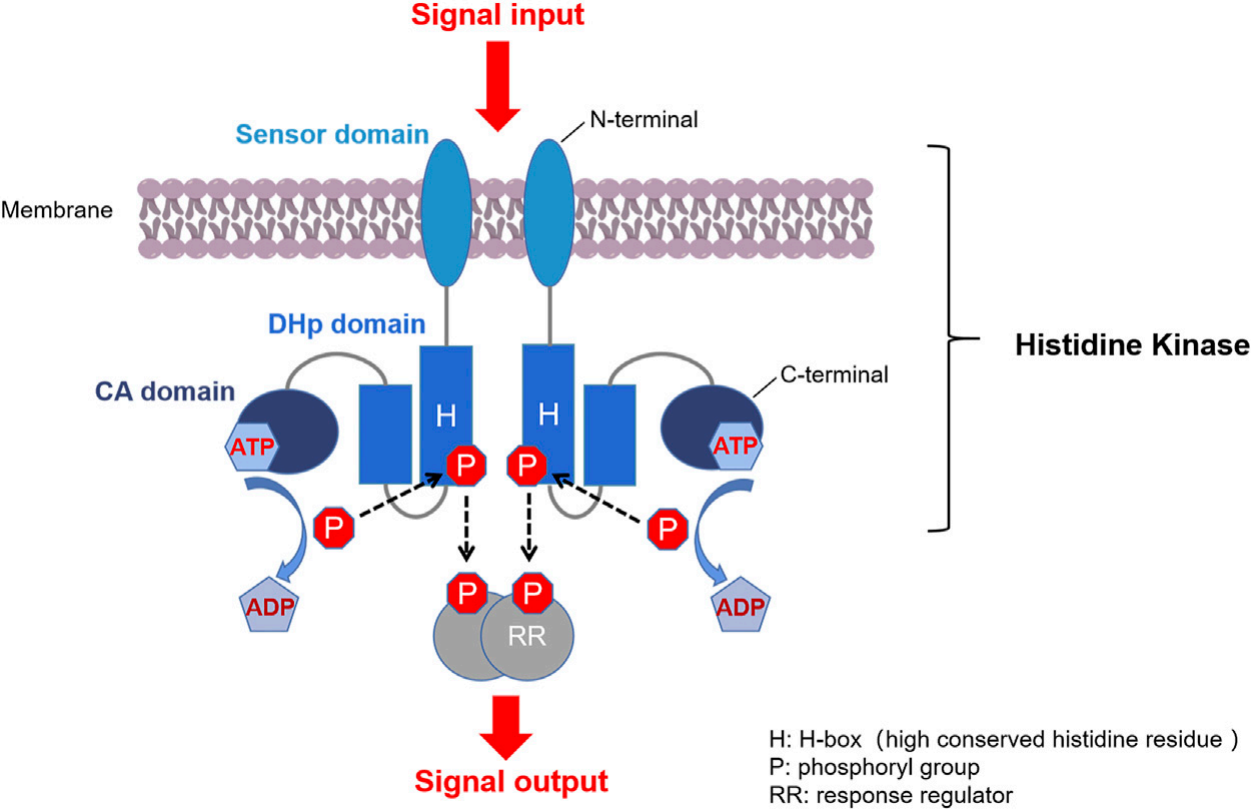
Typical TCS signaling pathway. First, a membrane-bound HK homodimer senses the signal and is auto-phosphorylated by ATP at the histidine residue. Second, the phosphoryl group on the phosphorylated HK is transferred to the conserved aspartate at the receiver domain of the cognate RR. Third, the phosphorylated RR (P-RR) interacts with target genes or proteins, and intracellular responses are triggered accordingly. Finally, P-RR is dephosphorylated by modulation of intrinsic or HK-induced P-RR autophosphatase activity (Stock et al., 2000; Casino et al., 2009).

Two-component systems (TCSs) are major mediators of signal transduction that exist ubiquitously in bacteria. They enable cells to sense, respond and adapt to a wide range of environmental conditions. Quite a few of them are also found in archaea, algae, fungi, protozoa, and plants, but typical TCS is absent from higher organisms such as humans (Papon and Stock, 2019). A typical bacterial TCS consists of two proteins: a homodimeric membrane-bound sensor histidine kinase (HK) and a cognate response regulator (RR) (Figure 1) (Casino et al., 2010; Bem et al., 2015). Most HKs contain a variable N-terminal extracellular sensor domain which is connected to a conserved C-terminal cytoplasmic kinase domain *via* transmembrane helices. RRs usually consist of a highly conserved N-terminal receiver domain, at which an aspartate functions as the acceptor of a phosphoryl group from the cognate HK, and a C-terminal effector domain that interacts with the downstream targets (Casino et al., 2009).

Advances in genome sequencing and gene function analysis have enabled the characterisation of various TCSs in bacterial species (Table 1). For example, 17 TCSs have been identified in the core genome of *Staphylococcus aureus* strains (Rajput et al., 2021); 29 HKs, 32 RRs, and one histidine-containing phosphotransfer domain (HPt) have been found from the complete genome sequence of *Escherichia coli* K12 (Mizuno, 1997); and 12 paired TCSs have been annotated in *Mycobacterium tuberculosis* H37Rv (Parish, 2014; Zheng and Abramovitch, 2020).

**TABLE 1:** The number of bacterial TCSs, HKs, and RRs in important human pathogens (Rajput et al., 2021).

Organism	The number of bacterial TCSs	The number of HKs, Signal transduction proteins (2022)	The number of RRs, Signal transduction proteins (2022)
*Staphylococcus aureus*	17, Rajput et al. (2021)	17	17
*Escherichia coli*	29, Rajput et al. (2021)	30	32
*Pseudomonas aeruginosa*	39, Rajput et al. (2021)	63	73
*Enterococcus faecium*	14, Rajput et al. (2021)	—	—
*Acinetobacter baumannii*	18, Rajput et al. (2021)	17	18
*Klebsiella pneumonia*	30, Rajput et al. (2021)	32	33
*Enterobacter* spp.	21, Rajput et al. (2021)	30	29
*Mycobacterium tuberculosis*	12, Parish (2014); Zheng and Abramovitch (2020)	13	13

Available evidence indicates that different TCSs and their corresponding HKs play respective roles in the regulation of the important life processes of bacteria and allow bacteria to adapt to extracellular physical/chemical stress (Tierney and Rather, 2019). Studies based on transposon mutagenesis and subsequent gene-functional validation revealed that most of TCSs were involved in regulating cell envelope biogenesis or cell division in pathogenic bacteria, and some of them were essential for cell growth and viability (Table 2). To date, at least nine essential TCSs related to the regulation of cell envelope-associated functions have been discovered from eight bacterial species, including CckA/CtrA from *Caulobacter crescentus*, WalK/WalR (also known as YycF/YycG) from *Bacillus subtilis* and *S. aureus* (Martin et al., 1999), AirS/AirR from *S. aureus*, VicK/VicR from *Streptococcus mutans*, PdhS/DivK from *Brucella abortus*, CprR/CprS from *Campylobacter jejuni*, MtrA/MtrB and PrrA/PrrB from *M. tuberculosis*, and EsaS/EsaR from *Burkholderia cenocepacia* (Van der Henst et al., 2012; Cardona et al., 2018).

**TABLE 2:** Essential TCSs in bacteria (Cardona et al., 2018).

Organism	Essential TCS	Physiological function
*Bacillus subtilis*	YycFG, Fabret and Hoch (1998)	Cell wall metabolism, Bisicchia et al. (2007), modulation of the expression of ftsAZ operon, Fukuchi et al. (2000)
*Brucella abortus*	PdhS/DivK, Hallez et al. (2007)	Cell cycle progression, Van der Henst et al. (2012)
*Burkholderia cenocepacia*	EsaSR, Gislason et al. (2017)	Integrity of cell membranes, antibiotic resistance, Gislason et al. (2017)
*Campylobacter jejuni*	CprRS	Biofilm formation, colonization and stress tolerance, Svensson et al. (2009)
*Caulobacter crescentus*	CckA-CtrA, Quon et al. (1996)	Cell cycle including cell division, stalk synthesis, and cell cycle-specific transcription, Quon et al. (1996), cell wall metabolism, Laub et al. (2002)
*Staphylococcus aureus*	WalKR(YycFG), Dubrac and Msadek (2004)	Cell permeability, Martin et al. (1999), cell wall metabolism and biofilm formation, Dubrac et al. (2007), cell division, Delaune et al. (2011); Hardt et al. (2017)
	AirSR(YhcSR)	Modulation of cell wall biosynthesis, susceptibility to vancomycin, Sun et al. (2013), nitrate respiratory pathway under anaerobic conditions, Yan et al. (2011)
*Streptococcus mutans*	VicKR	Cell wall synthesis, Ng et al. (2004)
*Mycobacterium tuberculosis*	MtrAB, Zahrt and Deretic (2000)	DNA replication and cell division, Parish (2014)
	PrrAB	Bacterial viability, regulation of nitrogen metabolism, Haydel et al. (2012)

One of the most remarkable examples of essential TCSs is WalK/WalR, which has been shown to be highly conserved among low GC gram-positive pathogens, including *S. aureus*, *Staphylococcus epidermidis*, *B. subtilis*, *Enterococcus faecalis*, *Streptococcus pneumoniae*, and *Streptococcus pyogenes* (Dubrac et al., 2008; Takada and Yoshikawa, 2018). Numerous studies have shown that WalK/WalR can coordinate cell wall metabolism during pathogen growth by sensing the products of peptidoglycan hydrolysis and regulating the expression and activity of the cell autolytic enzyme system in response (Dobihal et al., 2019). Another representative example of essential TCSs is the *M. tuberculosis* MtrA/MtrB, which conservatively exists in the genus *Mycobacterium* and many other high GC gram-positive bacteria, such as *Corynebacterium* spp., *Nocardia* spp., *Rhodococcus* spp., and *Streptomyces* spp. (Sarva et al., 2019). Studies have shown that MtrA controls DNA replication and cell division of *M. tuberculosis* by binding to the replication origin of *dnaA* and the promoter of the cell wall hydrolase-coding gene *ripA* to regulate their expression (Rajagopalan et al., 2010; Parish, 2014; Sarva et al., 2019). Although the MtrA/MtrB system is not essential in all mycobacteria, loss of the *mtrA* gene in *Mycobacterium smegmatis* results in defects in cell division and shape, and increasing susceptibility to some antimycobacterial drugs (Gorla et al., 2018), whereas the MtrB mutant of *M. smegmatis* displays a filamentous structure, defects in cell division, and a lysozyme-sensitive cell wall (Plocinska et al., 2012).

Besides a few essential TCSs, most TCSs, characterized as nonessential regulators, also play critical roles in physiological procedures (Table 3 The table contains some essential TCSs, which are also involved in regulating nonessential physiological processes). (Matsushita and Janda, 2002; Bem et al., 2015). Many nonessential TCSs are evolved to control a number of virulence factors, such as biofilms and quorum sensing (QS), in response to extracellular signals and stress (Worthington et al., 2013). For example, TCSs, such as AgrC/AgrA from *S. aureus*, PhoQ/PhoP from *E. coli*, and QseC/QseB from some gram-negative pathogens, including enterohemorrhagic *E. coli*, uropathogenic *E. coli*, and *Salmonella enterica* (Breland et al., 2017), have been shown to act as regulators of bacterial virulence and pathogenesis by modulating the processes of QS, biofilm/cell envelope formation, and/or virulence (Senadheera et al., 2005; George and Muir, 2007; Weigel and Demuth, 2016; Breland et al., 2017; Zhang et al., 2019; Yadavalli et al., 2020). Another TCS DosS-DosT/DosR from *M. tuberculosis* is involved in sensing host stimuli and triggering/maintaining dormancy (Roberts et al., 2004; Ioanoviciu et al., 2009). There are many other nonessential TCSs that can initiate a variety of antibiotic-resistance mechanisms, including cell surface modification, increased efflux, decreased influx, biofilm formation, and antibiotic-degrading enzyme induction (Tierney and Rather, 2019). For example, VanR/VanS regulates inducible vancomycin resistance in *Enterococcus faecium* by controlling the gene responsible for the synthesis of depsipeptide peptidoglycan precursors (Arthur et al., 1992). Similarly, VraR/VraS participates in the resistance of *S. aureus* to cell wall-acting antibiotics, such as glycopeptides and β-lactams, by mediating responses to the disruption of the early/late stages of cell wall biosynthesis (Worthington et al., 2013). Currently, it is believed that suppressing the virulence or antimicrobial resistance but not killing the pathogens could be a feasible strategy to develop new antibacterial drugs and may benefit the long-term clinical availability. Therefore, those biomolecular targets including TCSs affecting the virulence and resistance of bacteria have attracted high attention in recent years.

**TABLE 3:** TCSs implicated in bacterial virulence and antibiotic resistance (Stephenson and Hoch, 2002; Bem et al., 2015; Bhagirath et al., 2019; Tierney and Rather, 2019).

Pathogens	TCSs	Regulations	Antibiotic resistances
*Staphylococcus aureus*	ArlS/R	Regulates gene *norA* involved in drug efflux	Fluoroquinolone
		Regulates adhesion, autolysis, and extracellular proteolytic activity	
	AgrCA	Temporal expression of cell surface and secreted virulence factors in response to cell density	—
	BraSR	Upregulates MDR efflux pumps	Bacitracin, nisin
	GraRS	Modifies teichoic acids *via* D-alanylation which reverses bacterial surface charge	Daptomycin, vancomycin, cationic antibiotics
		Upregulation of VraFG ABC transporter	
	SaeRS	Produces over 20 virulence factors including hemolysins, leukocidins, superantigens, surface proteins, and proteases, Liu et al. (2016)	—
	SrrAB	Senses and responds to host-derived nitric oxide and hypoxia, Kinkel et al. (2013)	—
		Regulates globally virulence factor expression in response to environmental oxygen levels	
	VanSR	Alters vancomycin target through several actions	Vancomycin
	VraRS	Increases peptidoglycan synthesis and expression of PBP2	Methicillin, vancomycin, daptomycin, oxacillin, β-lactams
	WalKR (YycFG)^a^	Mechanism unknown; hypothesized increased permeability or decreased efflux	Macrolides, lincosamides, intrinsic resistance
		Increases formation of biofilm	
*Klebsiella pneumoniae*	PhoBR	Decreases porin expression	Tetracycline, nalidixic acid, tobramycin, streptomycin and spectinomycin
	CpxAR	Impacts membrane integrity, Raivio et al. (2013)	β-lactams, chloramphenicol
		Decreases porin expression	
		Upregulates MDR efflux pumps	
	EvgAS	Upregulates MDR efflux pumps	Intrinsic resistance
	PhoPQ	Modifies lipid A by 4-aminoarabinose, phosphoethanolamine (*via* PmrAB), or palmitate (*via* PagP)	Polymyxins
	PmrAB	Modifies lipid A by 4-aminoarabinose or phosphoethanolamine	Polymyxins
	RcsCB	Takes part in the capsular polysaccharide biosynthesis	—
		Regulates the production of major pilin protein MrkA	
		Confers resistance to low pH	
	QseC/B	Regulates the flagella and motility genes	—
*Acinetobacter baumannii*	AdeSR	Upregulates AdeAB(C) efflux pump	Aminoglycosides, fluoroquinolones, tetracycline, chloramphenicol, erythromycin, trimethoprim, intrinsic resistance
	BaeSR	Upregulates MDR efflux pumps	Tannic acid
	BfmRS	Increases formation of biofilm	Chloramphenicol, intrinsic resistance
	GacSA	Increases formation of biofilm	Intrinsic resistance
	PmrAB	Regulates genes involved in lipopolysaccharide modification	Polymyxins
*Pseudomonas aeruginosa*	CzcRS	Decreases porin expression	β-lactams, carbapenems
	AmgRS	Upregulates MDR efflux pumps, activates stress response protects the membrane	Aminoglycosides, intrinsic resistance
	CopRS	Decreases porin expression	β-lactams, carbapenems
	CprRS	Modifies lipid A by 4-aminoarabinose (*via arn* operon)	Polymyxins, aminoglycosides
	CreBC	Activates β-lactamase gene, increases formation of biofilm	β-lactams, intrinsic resistance
	EvgAS	Upregulates MDR efflux pumps	Intrinsic resistance
	ParRS	Modifies lipid A by 4-aminoarabinose (*via arn* operon)	Polymyxins, aminoglycosides
	RetS-GacSA	Increases formation of biofilm	Intrinsic resistance
	SagS	Upregulates MDR efflux pumps, increases formation of biofilm *via* BfiSR TCS	Tobramycin, Intrinsic resistance
	PhoPQ	Modifies lipid A by 4-aminoarabinose, phosphoethanolamine (*via* PmrAB), or palmitate (*via* PagP)	Polymyxins, tetracycline, intrinsic resistance
		Regulates ABC transporter system and drug efflux, Chen and Duan (2016)	
*Mycobacterium tuberculosis*	DosRST	Promotes nonreplicating persistence, Zheng and Abramovitch (2020)	—
	MtrAB^a^	Upregulates efflux pumps	Multidrug
	PhoPR	Regulates intracellular growth in macrophages	—
	PrrAB^a^	Implicated in adaptation to the phagosome environment in macrophages	—
*Enterococcus* species	CroRS	Upregulates PBP5	Ceftriaxone (β-lactam)
	FsrC/FsrA	Increases formation of biofilm *via* production of gelatinase	Intrinsic resistance
		Takes part in cell density signaling	
	VanSR	Controls the genes responsible for the synthesis of the modified peptidoglycan precursors	Vancomycin
*Salmonella enterica*	PhoPQ, PmrAB	Regulates lipid A vickstructure and acidic glycerophospholipid	Antimicrobial peptide, polymyxin
	BaeSR	Upregulates MDR efflux pumps	Ciprofloxacin
*Escherichia coli*	BaeSR	Upregulates MDR efflux pumps	Novobiocin, deoxycholate
	CheAY	Mediates chemotaxis towards the amino acid attractant aspartate, Kim et al. (2001)	—
	CpxAR	Decreases porin expression	Chloramphenicol, amikacin, nalidixic acid, tetracycline
		Upregulates MDR efflux pumps	
	ArcBA	Mediates anaerobic expression of the *gadE-mdtEF* multidrug efflux operon	Multidrug
	EnvZ/OmpR	Decreases the expression levels of outer membrane porin proteins	β-lactams
	EvgAS	Upregulates MDR efflux pumps	Intrinsic resistance
	BaeSR	Regulates the expression level of outer membrane proteins. Up-regulates the expression of drug exporter genes	β-lactams, novobiocin
	RcsBCD	Senses surface contact	—
		Takes part in colanic acid capsule synthesis, Vianney et al. (2005)	
*Stenotrophomonas maltophilia*	SmeSR	Upregulates SmeZ efflux pump	Aminoglycosides, β-lactams,fluoroquinolones
*Streptococcus* species	CiaRH	Development of genetic competence	Cefotaxime
	LiaSR	Regulates cell wall stress responses	Vancomycin, bacitracin, nisin, chlorhexidine
		Regulates virulence traits such as acid tolerance and biofilm formation	
		Induces genes encoding PG synthesis and remodeling enzymes in addition to membrane protein synthesis and envelope chaperone/proteases, Suntharalingam et al. (2009)	
*Bacillus subtilis*	ResE-ResD	Regulates aerobic and anaerobic respiration, Zhang and Hulett (2000)	—
*Caulobacter crescentus*	DivJ-DivK	Regulates cell differentiation and division, Wu et al. (1998)	—

a essential HKs, which also participates in regulating bacterial virulence or antibiotic resistance.

Overall, the crucial roles of many TCSs are not only in the bacterial growth and viability, but also in regulating essential pathogenic processes such as the virulence factors production, biofilm formation, and antibiotic resistance, which highlighted the importance of these TCSs as attractive drug targets. In contrast to conventional antibiotics that primarily target specific functional proteins essential for viability of the pathogens, a drug that targets TCSs could exert its potentially antibacterial effects by impairing upstream regulatory functions related to the pathogen’s diverse physiological processes like intracellular persistence, pathogenicity, and metabolism. Therefore, the use of TCSs for drug development offers an alternative strategy to combat bacterial infections, including those caused by pathogens that are resistant to currently available antibiotics (Tiwari et al., 2017). Moreover, the mechanism of signal transduction in eukaryotes involves multiple phosphotransfers between His- and Asp-containing proteins, which is different from bacteria. The TCS system is absent in human cells, therefore, TCSs are attractive targets for antibacterial treatments with low potential for toxicity in humans, so the development of specific TCS inhibitors could lead to novel antimicrobial agents (Thomason and Kay, 2000; Matsushita and Janda, 2002; Gao and Stock, 2009).

### 1.3 Histidine Kinase as Targets for Novel Antimicrobial Agents

**FIGURE 2:**
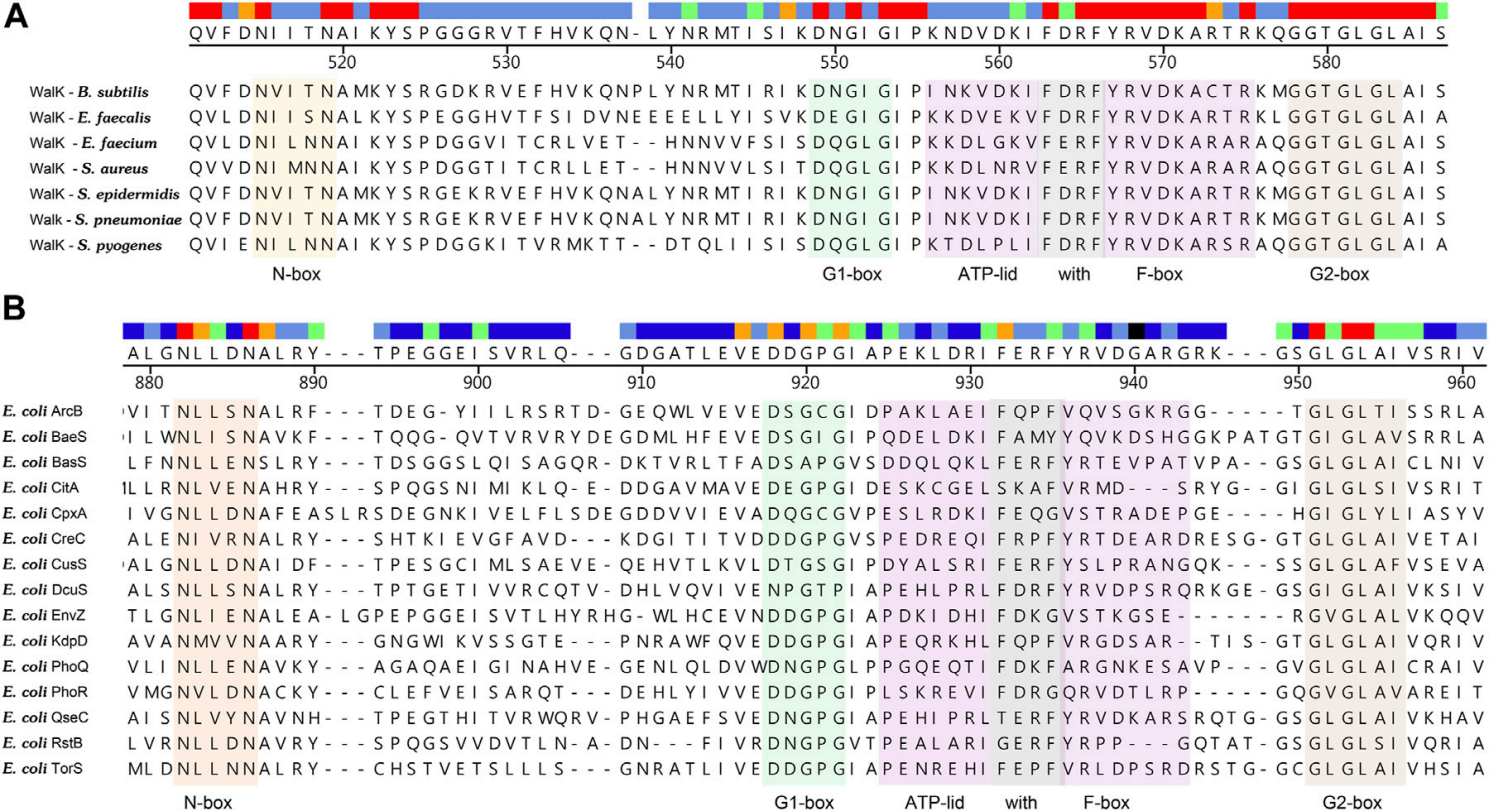
**(A)** Sequence alignment of CA domains of WalK from seven important gram-positive pathogens. The N-box, G1-box, G2-box, F-box, and ATP-lid are colored in orange, green, light brown, gray, and purple, respectively. The high conservation of CA catalytic site indicated that inhibitors targeted at this site will possess broad-spectrum antibacterial activity. **(B)** Sequence alignment of CA domains of fifteen kinds of HKs from *E. coli*. High sequence similarities of the CA domain from different HKs implied that a HK inhibitor acting on multiple targets is a promising strategy to slow down antibacterial resistance (Bem et al., 2015).

Although the sensor and effector domains and the regulatory mechanisms of different TCSs are variable, the catalytic ATP-binding domain (CA) and dimerization and histidine phosphorylation (DHp) domains of HKs are conserved (Gao and Stock, 2009), which lead the possibility to design broad-spectrum antimicrobial agents. Sequence alignment of CA domains of WalK from seven important gram-positive pathogens and sequence alignment of CA domains of fifteen kinds of HKs from *E. coli* were shown in Figure 2.

**FIGURE 3:**
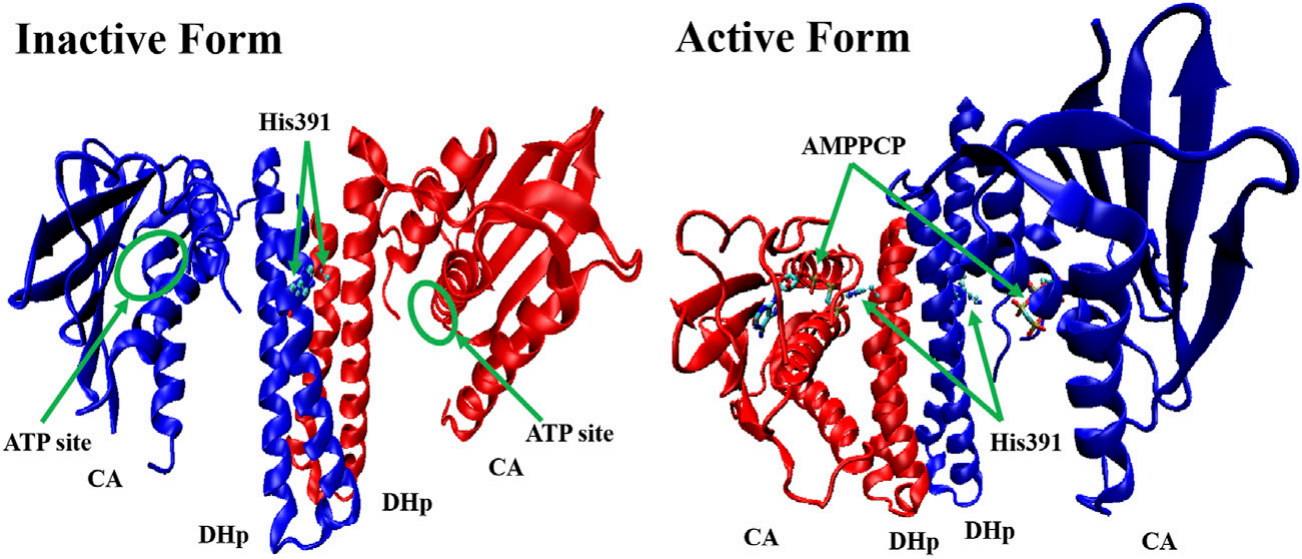
The crystal structure of inactive form (left, PDB: 4U7N) and active form (right, PDB: 5C93) of WalK (Cai et al., 2017). WalK is represented by a cartoon tube model, the His391 is represented by balls-sticks model, and AMPPCP (a nonhydrolyzable analog of ATP) is represented by sticks model, in which oxygen atoms are colored in red, nitrogen atoms are colored in blue, carbon atoms are colored in cyan, and phosphate atoms are colored in tan.

All HKs share the same core region (∼250 amino acids), which contains DHp and CA (Cai et al., 2017). Studies of the crystal structures of *Lactobacillus plantarum* WalK clearly indicate that the HK contains inactive (in the absence of ATP or ADP) and active (ATP or ADP binding) forms. In the absence of ATP or ADP, as shown in Figure 3, WalK is in a symmetric open status, in which two DHp domains are located in the centre and two CA domains are outside like two wings and the ATP binding sites (on CA domains) are far from key residue His391 (on each DHp domain). Consequent to the binding of ATP, WalK adopts a major conformational change *via* the movement of one CA domain closing to the DHp domain to form an asymmetrical closed conformation. In the active form, the phosphate group of ATP moves close to His391, enabling the phosphorylation on His391 to occur.

**FIGURE 4:**
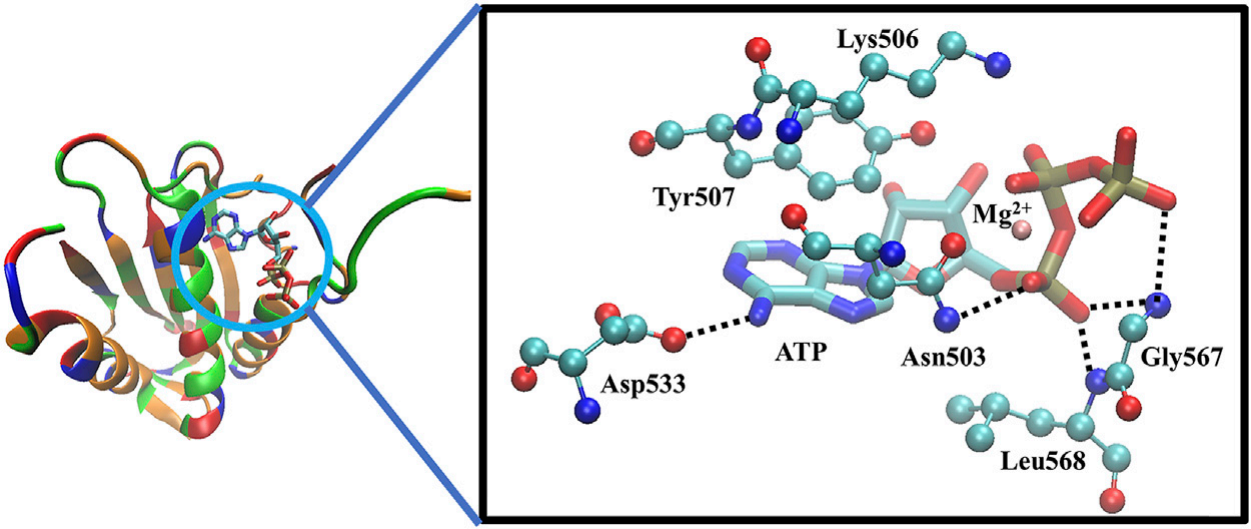
The X-ray crystal structure of the ATP-binding domain of WalK (PDB: 3SL2) (Celikel et al., 2012; Boibessot et al., 2016). Left: the overview of the binding of ATP with WalK, in which the ATP is represented by sticks model and Walk is represented by a cartoon tube in which the basic residues are colored in blue, the acidic residues are colored in red, the polar residues are colored in green and the nonpolar residues are colored in orange. Right: The binding interactions between ATP (balls and sticks model) and the key residues on WalK (sticks model). Oxygen atoms are colored in red, nitrogen atoms are colored in blue, carbon atoms are colored in cyan, phosphate atoms are colored in tan, and Mg^2+^ are colored in orange. Hydrogen bonds are represented by dotted lines, and chelation with Mg^2+^ is represented by orange lines.

The CA domain of HKs is a popular target in recent drug discovery studies and contains a highly conserved ATP-binding region between bacteria and eukaryotic proteins, characterized by a Bergerat fold, which is a common domain shared with DNA-gyrases and topoisomerase, heat shock proteins 90 (Hsp90), and the mutator protein MutL (Boibessot et al., 2016). Because of such similarity, a potential strategy to discover new HK inhibitors was established, based on the hypothesis that Hsp90 inhibitors, which have been investigated as anticancer drugs, could also interact with the ATP-binding pocket of HKs and block autophosphorylation (King and Blackledge, 2021). For example, a series of diaryl pyrazole compounds were discovered as PhoQ inhibitors by screening and modifying the known Hsp90 inhibitors (Vo et al., 2017). However, the high similarity also caused inhibitors’ lack of selectivity on multiple TCSs (Wilke and Carlson, 2013; Wilke et al., 2015), which should be considered during the drug design process to prevent off-target inhibitory effects and toxicity (Guarnieri et al., 2008; Bem et al., 2015). The cocrystal structure of the ATP in complex with CA of WalK has been solved as Figure 4 shows, in which ATP binds between α-helices and β-strands, and a flexible loop (Asp534-Gly567) mediates the ATP’s binding by enabling the conformational changes to occur to facilitate the binding of ATP. The crystal structure clearly shows that the ATP forms direct hydrogen bonds with Asp533, Asn503, Leu568, Gly567, Leu566, and Gly565. Together with Asn503, ATP forms the chelation with the Mg^2+^, Tyr507 forms π-π interactions, and Lys506 forms strong electrostatic interactions with ATP.

Another important direction for the discovery of new HK inhibitors is to design inhibitors by targeting the DHp domain, which is formed by two long helices (α1 and α2), where α1 contains a phosphorylatable conserved His residue (His391, as shown in Figure 3) (Casino et al., 2014). This domain contains His391 is called H-box (shown in Figure 1, the position of the histidine phosphorylation site is marked with an “H” indicating the H-box region), which is the site of autophosphorylation (Wang et al., 2013; Kato et al., 2017). This structural homology of HKs indicates the possibility that multiple TCSs can be inhibited simultaneously by one inhibitor, and decrease the appearance of drug-resistant strains.

Overall, intrinsic characteristics make HK proteins attractive targets for the identification of new antibacterial agents. Firstly, HKs are embedded in many pathogens, and some of them are essential for bacterial survival or play important roles in mediating virulence or antibiotic resistance (Matsushita and Janda, 2002). Secondly, the key residues in the CA and DHp domains contain a high level of structural conservation and sequence homology, which is a key for the design of novel broad-spectrum HK inhibitors (Bem et al., 2015). Thirdly, mammalian genomes do not contain HK proteins, while other types of kinases, such as serine/threonine kinases and tyrosine kinases, share low structural homology with HK proteins, by which specific HK inhibitors may show little toxicity in mammalian cells (Casino et al., 2009; Rosales-Hurtado et al., 2020). Finally, as HK is not a traditional drug target for conventional antibiotics, such inhibitors are not influenced by the development of cross-drug resistance in multiple drug resistance (MDR) pathogens (Hirakawa et al., 2020).

## 2 Novel Histidine Kinase-Targeted Antimicrobial Agents

In recent years, with a more in-depth understanding of the structure, function, and physiological importance of bacterial HKs, more and more small-molecule HK-targeted inhibitors with good antibacterial activity and medicinal potential have been discovered through molecular screening and design strategies. Herein, we have collected and analysed the antibacterial data reported in the literature in the last 10 years, focusing on the latest research progress of small-molecule antimicrobial agents targeting HKs. The small-molecule inhibitors described below target at different domains of HKs, such as CA domain, DHp domain, or sensor domain, while the specific targets of some HK inhibitors have not yet been elucidated. The current review summarizes and emphasizes the studies on the characterization of enzyme-inhibition and antibacterial activity of novel HK inhibitors, which will be helpful for the future discovery of new antimicrobial agents (Table 4).

**TABLE 4:** The summary of HK inhibitors.

Target	Structure classification	Structure	The half-maximal inhibitory concentration (IC_50_, μM)	The minimum inhibitory concentration (MIC)	Antivirulence
CA domain	LUT	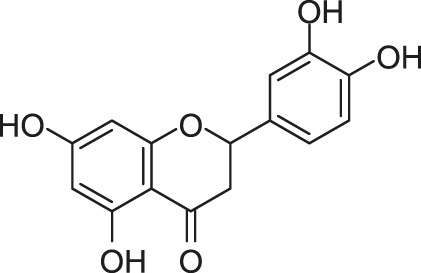	HK853: 15.1	*E. coli:* 8 μg/ml	—
			VicK: 216		
			CheA: 111		
	Benzothiazole	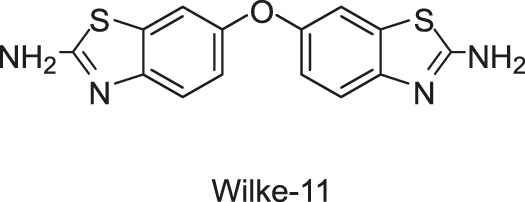	HK853: 1.21	*B. subtilis:* 49–64 μg/ml	—
			VicK: 75	*E. coli*: 32–64 μg/ml	
		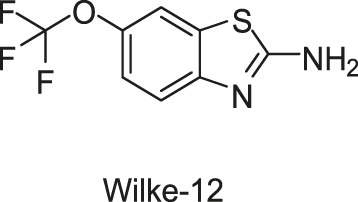	HK853: 7.15	*B. subtilis:* 293 μg/ml	—
			VicK: 618		
			CheA: 1,340	*E. coli*: 128 μg/ml	
		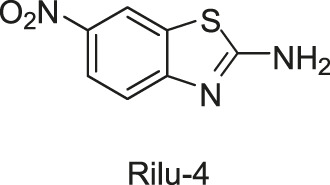	HK853: 8.3	—	Reduce the production of virulence factors
		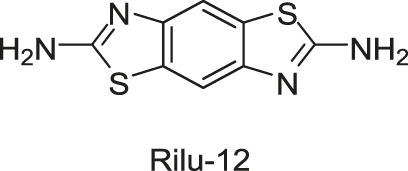	HK853: 1.56	—	
	Thiazolidione	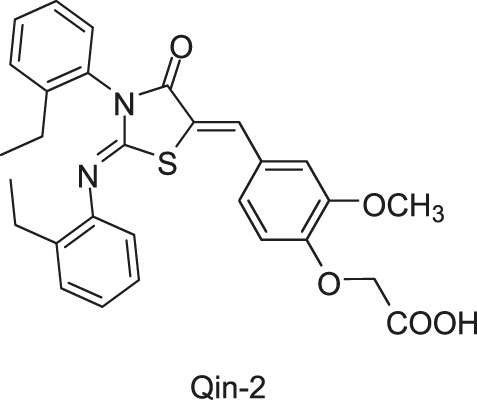	WalK: 29	—	—
		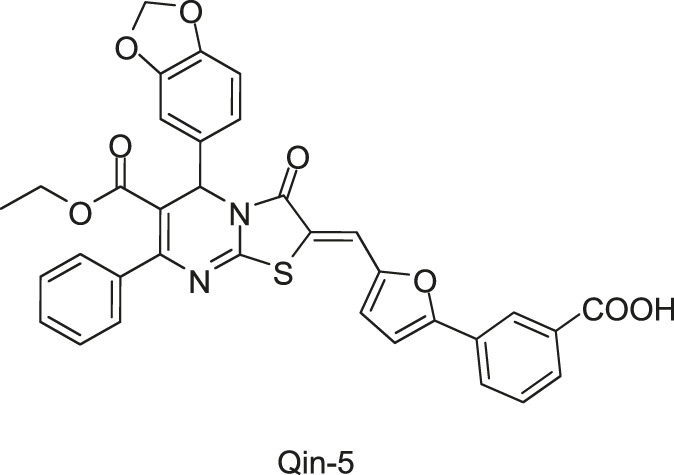	WalK: 14	—	—
		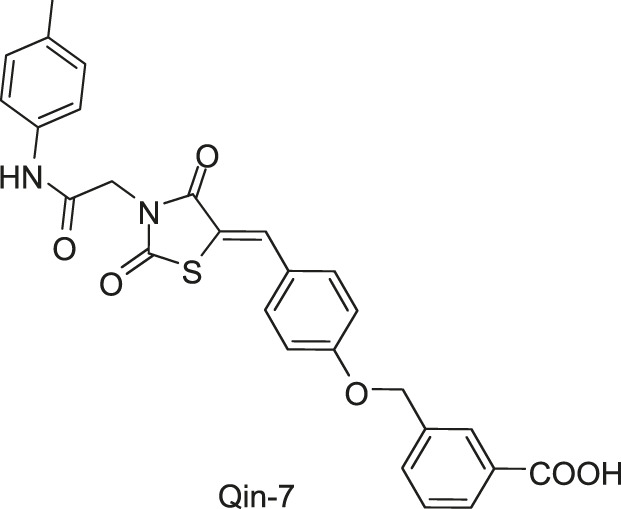	WalK: 6.5	—	—
		Qin-2 derivatives (Pan-10, -12, etc., Figure 5)	WalK: 22.15–88.35	*S. epidermidis*: (Pan-12, -20, -27, -28, Huang-29) 1.5–6.6 μg/ml, (Pan-10) 12.2 μg/ml	—
		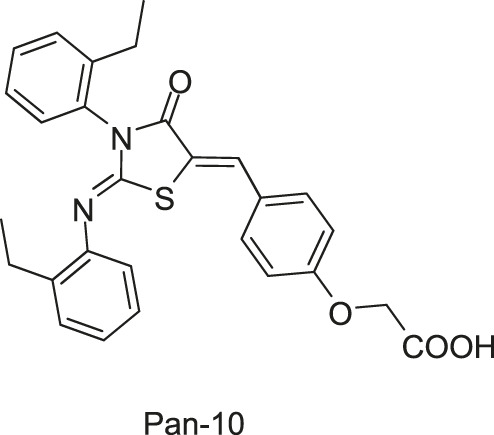		*S. aureus*: (Pan-12, -20, -27, -28, Huang-29) 1.5–6.6 μg/ml, (Pan-10) 24.3 μg/ml	
		Qin-2 derivatives (Liu-38, -39, etc., Figure 6)	WalK: 24.2–71.2	*S. epidermidis* and *S. aureus*: 1.5–6.3 μM	Antibiofilm activities against *S. epidermidis*
		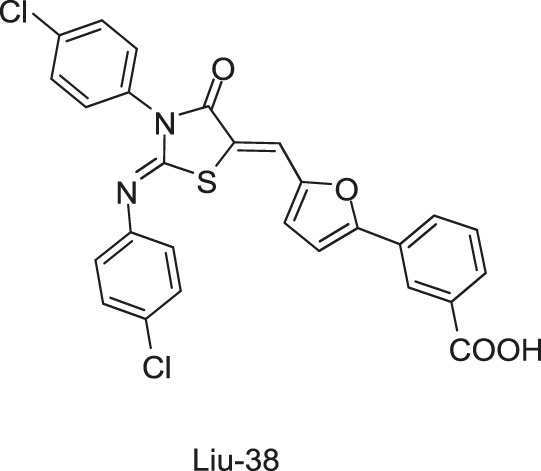			
		Qin-5 derivative (Zhao-4e) 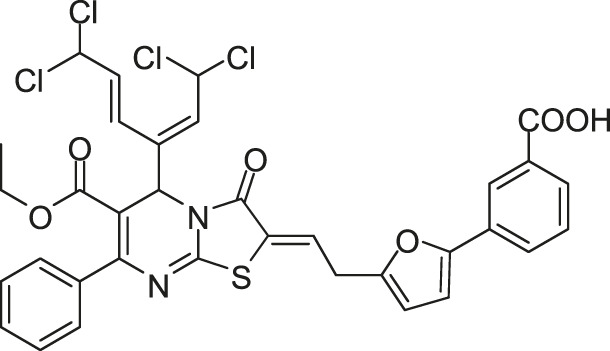	WalK: 12.65	*S. epidermidis* and *S. aureus*: 1.143–4.578 μg/ml	Antibiofilm activities against *S. epidermidis*
		Qin-5 derivatives (Lv-23, -24, etc., Figure 7)	WalK: 61.94–83.91	*S. epidermidis* and *S. aureus*: 1.56–6.25 μM	—
		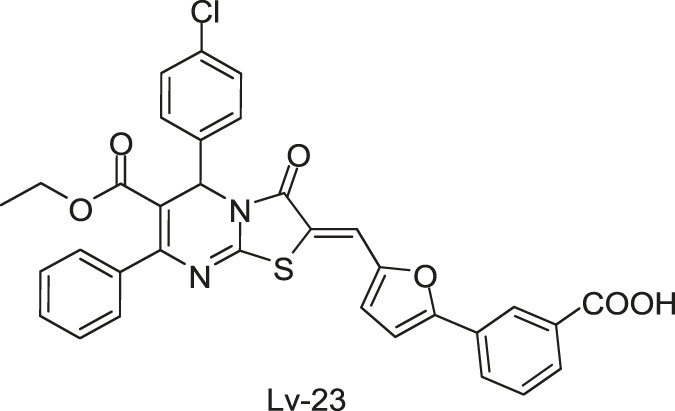			
	Thiophene	Boibessot-6d, -6e, etc., Figure 8	PhoR: 1.63–122.6	*B. subtilis* and *Bacillus anthracis:* 7 μg/ml	Be used as an adjuvant when combining with amoxicillin or cefotaxime, and restores the sensitivity of resistant isolates
		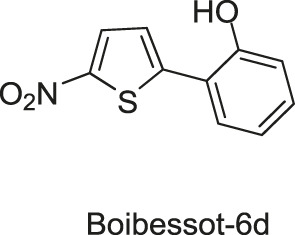	ResE: 20.3–243.9	*S. aureus*, *S. pyogenes*, *L. monocytogenes*, *S. enterica*, and *E. coli*: 10–32 μg/ml	
	Diaryl pyrazole	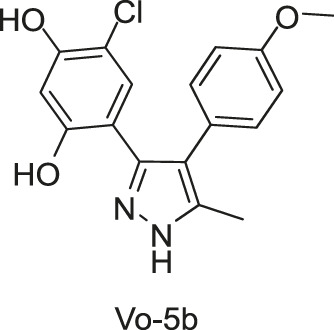	CckA: 28	*C. crescentus:* 74.4 μg/ml	—
				*B. subtilis:* 49.6–74.4 μg/ml	
			PhoQ: 238	*E. coli*: 12.4–24.8 μg/ml	
	Velikova-13	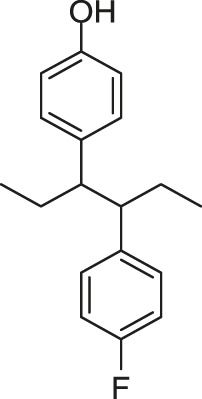	PhoR of *S. aureus*: 212	*S. aureus:* 8–16 μg/ml	—
				*S. epidermidis:* 1–16 μg/ml	
			PhoR of *E. coli*: 16	*S. pneumoniae*: 16 μg/ml	
	Five traditional Chinese medicine monomers	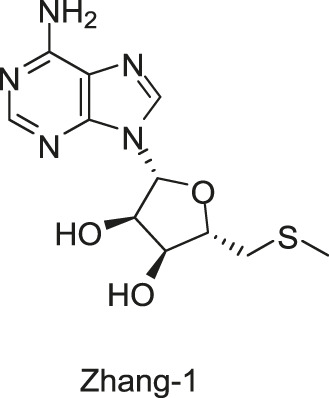	VicK: 3.8	*S. pneumoniae*: 37.1 μg/ml	Inhibit the formation of *S. pneumoniae* biofilm
		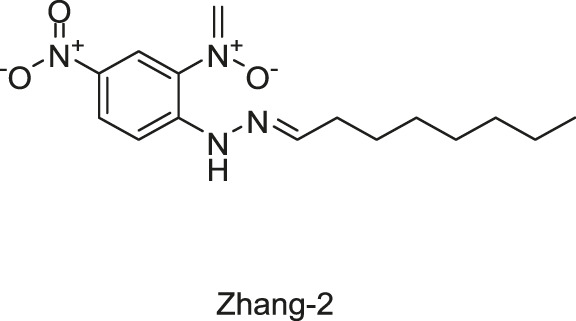	VicK: 5.4	*S. pneumoniae*: 38.5 μg/ml	
		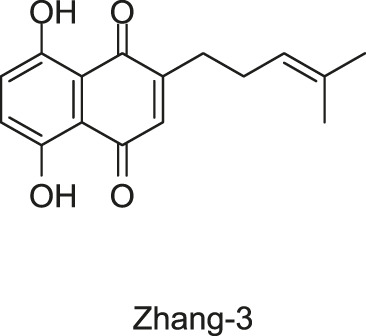	VicK: 15.4	*S. pneumoniae*: 17 μg/ml	
		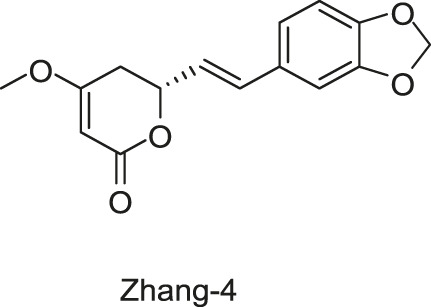	VicK: 4.6	*S. pneumoniae*: 68.5 μg/ml	
		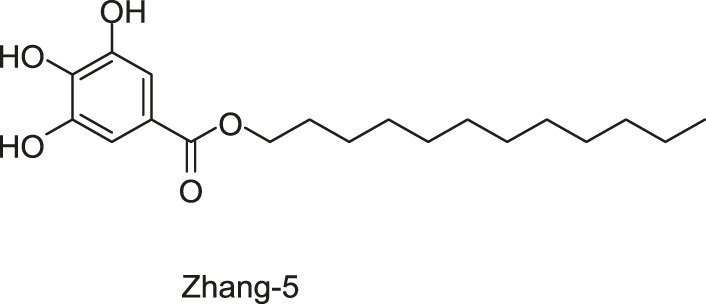	VicK: 9.1	*S. pneumoniae*: 21 μg/ml	
DHp domain	Signermycin B	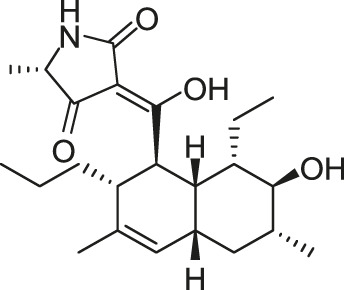	WalK: 37–62	Gram-positive bacteria: 3.13–6.25 μg/ml	—
	Waldiomycin	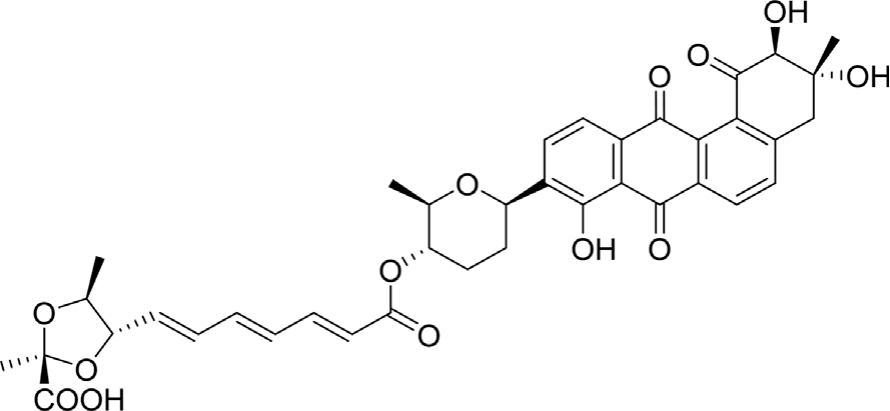	WalK: 8.8–25.8 QseC: 15.1 EnvZ: 22.4 PhoQ: 12.5	*S. aureus* and *B. subtilis*: 8–16 μg/ml	—
Sensor domain	Maprotiline	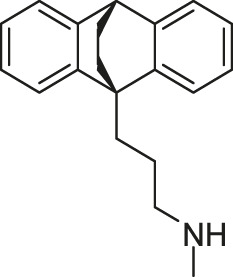	QseC: -	—	Decrease formation of biofilm; disrupt expression of TCS-dependent virulence factor
Inhibitor as a prodrug	LED209	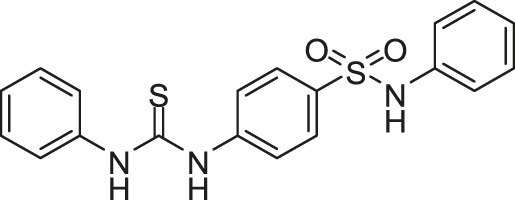	QseC:-	—	Inhibit QseC-mediated activation of virulence gene expression
					Decreases biofilm formation
Targets under validation	Walkmycin B	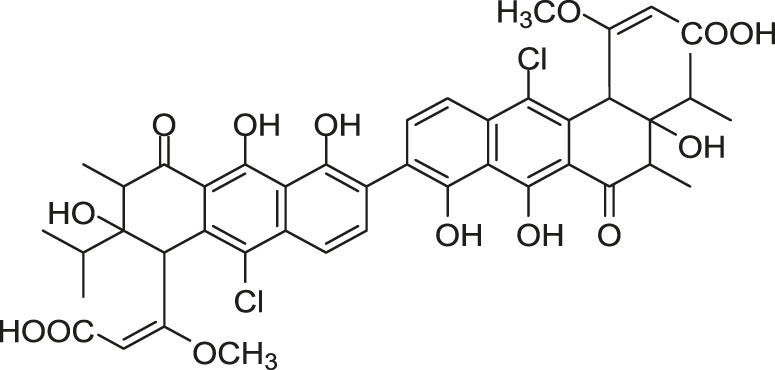	WalK of *B. subtilis*: 1.6	*B. subtilis*, *S. aureus*, and *E. faecalis*: 0.20–6.25 μg/ml	—
			WalK of *S. aureus*: 5.7		
	Walkmycin C	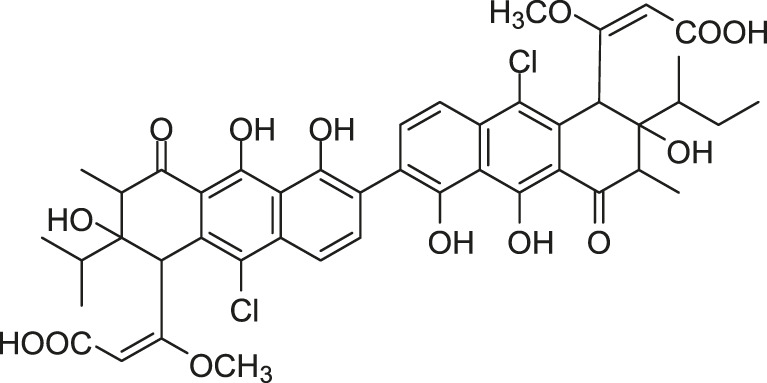	VicK: 2.87	*S. aureus*, *B. subtilis*, *E. faecalis*, *S. pneumoniae*, and *S. pyogenes*: 0.0625–16 μg/ml	—
			CiaH: 4.87		
			LiaS: 5.63		
			EnvZ: 1.25		
			PhoQ: 1.25		
	Rhein	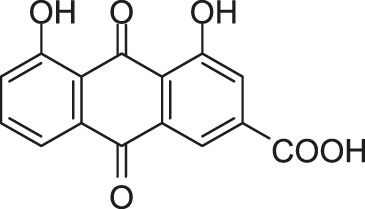	AgrC: 13.7	*S. aureus*: 32 μg/ml	Decrease formation of biofilm; reduce the expression of three virulence factors which were regulated by the agr system
	Aloeemodin	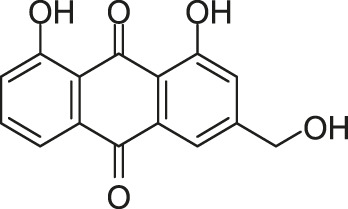	AgrC: 62.2	*S. aureus*: 64 μg/ml	
	Cai-1	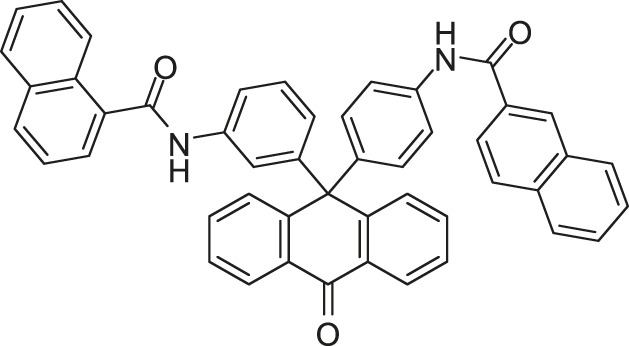	PhoQ: 69.37	—	Reduce the virulence of Shigella
	Cai-2	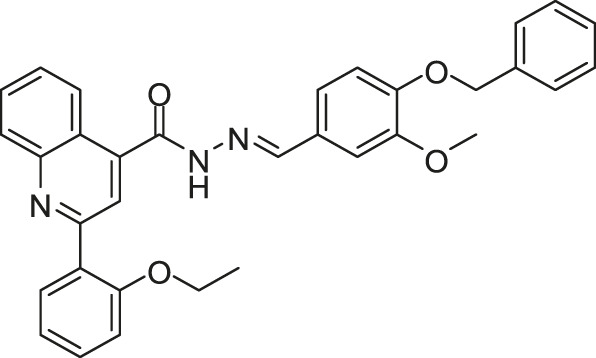	PhoQ: 48.9	—	
	Cai-3	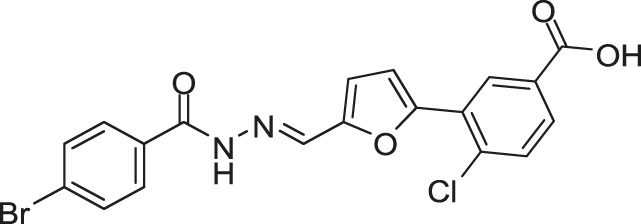	PhoQ: 7.99	—	
	Cai-4	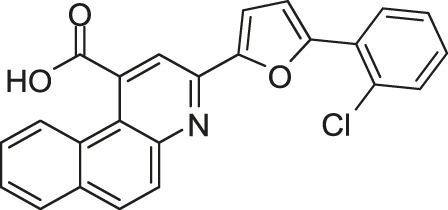	PhoQ: 27.2	—	
	Diarylthiazole derivatives	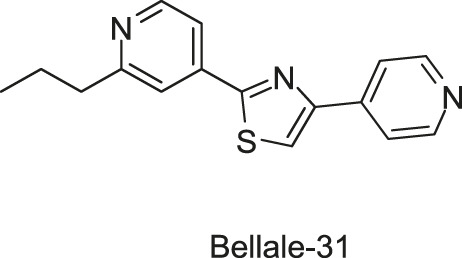	—	*M. tuberculosis*: 0.4 μg/ml	
		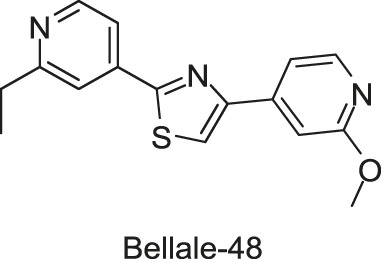	—	*M. tuberculosis*: 0.25 μg/ml	—
	Xanthoangelol B derivatives	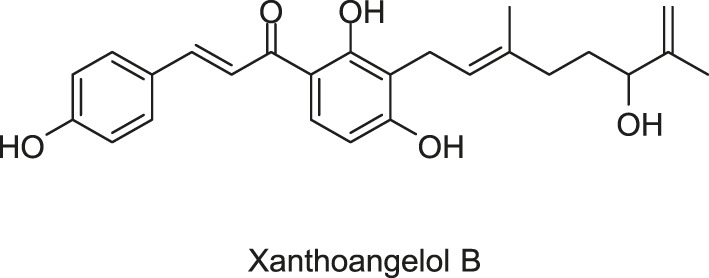	SaeS: 220	—	Suppress transcription of four downstream virulence genes (α-hemolysin (hla), aureolysin (aur), γ- hemolysin, and staphylokinase)
			AgrC: 339		
		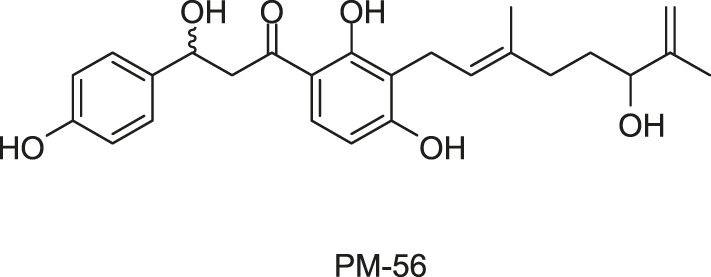	SaeS: 160		Suppress transcription of four downstream virulence genes (α-hemolysin (*hla*), aureolysin (*aur*), γ-hemolysin, and staphylokinase)
			AgrC: 140		
	Zheng-103A	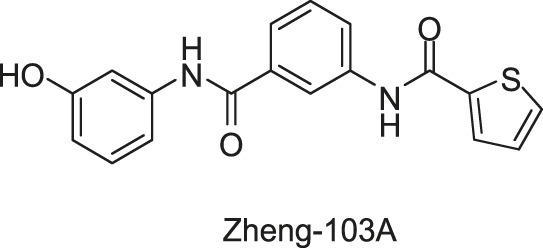	DosS: 0.5 DosT: 5	—	—
	Zheng-102A	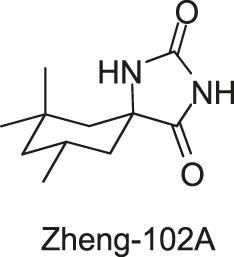	DosS: 1.9	—	—

### 2.1 Inhibitors Targeting the Catalytic ATP-Binding Domain

#### 2.1.1 Luteolin

To find small molecules with the broad anti-HK activity, Wilke et al. (2015) developed a fluorescence polarization displacement assay-based high-throughput screening (HTS) method and performed the screen of ∼53,000 diverse small molecules for their competitive binding to the ATP site of HKs. In this work, the researchers employed three conserved HKs (HK853 from *Thermotoga maritima*, VicK from *S. pneumoniae*, and CheA from *E. coli*) representing the 11 subfamilies of HKs as enzymatic inhibition targets and used ADP-BODIPY, a fluorescent nonhydrolyzable adenosine diphosphate (ADP) probe, as an indicator for ATP-competitive inhibitors of HKs in the screening. The natural product luteolin (3’,4’,5,7-tetrahydroxyflavone, also referred to as LUT) was discovered, which is a widely distributed flavonoid compound derived from vegetables, fruits, and herbs with a range of proposed mechanisms, including antioxidant, and anti-inflammatory activities.

LUT is structurally similar to the flavonoid genistein, which was previously reported to inhibit a yeast HK but only showed a slight inhibition on HK853 activity in follow-up assays (Huang et al., 1992; Wilke et al., 2015). In contrast to the flavonoid genistein, LUT inhibited HK853, VicK, and CheA with the half-maximal inhibitory concentrations (IC_50_s) of 15.1, 216, and 111 μM, respectively, and inhibited the growth of *E. coli* DC2 with a minimum inhibitory concentration (MIC) of 8 μg/ml, but showed no activity against *B. subtilis* 3610 (MIC >128 μg/ml), without significant cytotoxicity on Vero 76 cells (Wilke et al., 2015). Zhou et al. (2019) *,* by combining with Nuclear Magnetic Resonance (NMR), isothermal titration calorimetry (ITC), and molecular docking studies, it was predicted that LUT inhibits the autophosphorylation activity of HK853 from *Thermotoga maritime* (*T. maritime*) by occupying the binding pocket of ADP by forming hydrogen bonds and π-π stacking interaction. As the analysis of binding interactions was only based on molecular docking and has not been confirmed by any experimental data, in the current review, we do not include such information.

However, LUT is a well-known multiple targets binding antagonist with inhibition of fatty acid synthase, angiotensin, and vascular endothelial growth factor receptor. It also can bind to a Ser/Thr protein kinase named casein kinase 2(CK_2_) (Lolli et al., 2012; National Center for Biotechnology Information, 2022). Thus, the poor selectivity of LUT for HKs has limited its further development as a specific HK inhibitor.

#### 2.1.2 Benzothiazole

In the same study in which LUT was screened out, two benzothiazole compounds, compounds 11 and 12, were also obtained through the HTS method based on the fluorescence polarization displacement assay by Wilke et al. (2015). To distinguish the cited compounds in this review, they have been renamed by using the surname of the first author plus their original IDs. For example, the compounds 11 and 12 in the original paper were renamed as Wilke-11 and 12, respectively. All the compounds in this review, except those with specific names, are renamed according to this rule. Wilke-11 showed activities against HK853 and VicK, with IC_50_ values of 1.21 and 75 μM, respectively, and Wilke-12, the known US Food and Drug Administration (FDA)- approved medicine named riluzole for amyotrophic lateral sclerosis, was found to be active against HK853, VicK, and CheA, with IC_50_s of 7.15, 618, and 1,340 μM, respectively. Compared with Wilke-12, Wilke-11 exhibited increased HK inhibitory potency, suggesting that the aminobenzothiazole is possible to be an important scaffold in the development of HK inhibitors.

Wilke-11 inhibited the growth of *B. subtilis* 3610 and *E. coli* DC2, with MICs ranging from 49 μg/ml to 64 μg/ml and from 32 μg/ml to 64 μg/ml respectively, which is lower than that of Wilke-12 against the same bacteria (MIC = 293 and 128 μg/ml, respectively). However, these compounds were cytotoxic against Vero 76 cells at concentrations exhibiting antibacterial effects, which should be improved in further structural optimizations (Wilke et al., 2015).

In general, Wilke’s compounds provide a possible starting point for the design of broad-spectrum HK inhibitors and the aminobenzothiazole group is widely treated as a general scaffold for the following structural optimization in numerous bacterial species.

Subsequently, based on the leading compounds of Wilke-11 and 12, Goswami et al. (2018) generated a small library of compounds containing the benzothiazole as the centre core for HTS screening, and Rilu-4 and -12 were identified as the most promising compounds. Similar to Wilke-11, Rilu-12 contains a rigid tricyclic structure with two amino moieties, which performed an inhibitory effect with IC_50_ as 1.56 μM against HK853. Rilu-4 contains an electron-withdrawing group −NO_2_, and performed an IC_50_ of 8.3 μM against HK853. Moreover, Rilu’s compounds significantly reduced the production of virulence factors, such as the *Pseudomonas* quinolone signals (PQS) and toxins (e.g., pyocyanin, phenazine-1-carboxamide, and phenazine-1-carboxylic acid) which were involved in QS and redox-balancing mechanisms. They severely affected the motility behaviors of *P. aeruginosa* PA14 (A strain obtained from a burn wound, which can encode a large number of TCSs in its genome), thereby decreasing the ability to swarm and attach to surfaces. These benzothiazole derivatives provide the possibilities of applications as potential antivirulence agents, which is a viable alternative to traditional antibacterial therapy for the treatment of infections caused by antibiotic-resistant organisms, either alone or in combination with antibiotic treatment. At present, researchers are aiming to improve the efficacy and explore the key inhibition mechanisms of Rilu-inhibitors.

#### 2.1.3 Thiazolidione

Qin et al. (2006) firstly used a structure-based virtual screening (SBVS) method to discover potential inhibitors of the *S. epidermidis* WalK from a small molecular leading compound library, and among 76 candidates targeting the WalK ATP-binding domain. Seven compounds displayed significant growth inhibitory activity against *S. epidermidis*. Among them, Qin-2, -5, and -7 were thiazolidione analogs. Qin-2 and -5 were effective against *S. aureus*, *S. pyogenes*, and *S. mutans*, and Qin-7 was only effective against the non-biofilm-forming *S. epidermidis* ATCC 12228. All compounds inhibited the ATPase activity of WalK protein, with IC_50_s ranging from 6.5 to 29 μM.

**FIGURE 5:**
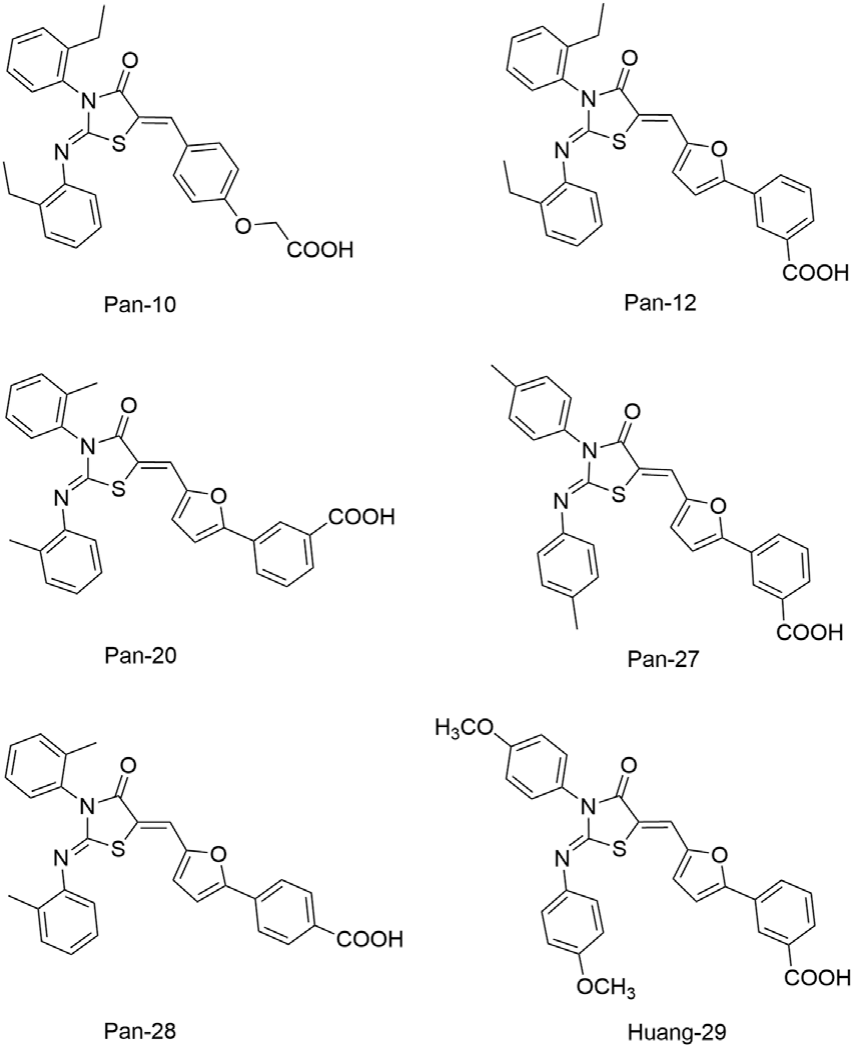
The chemical structures of Pan-10, Pan-12, Pan-20, Pan-27, Pan-28, and Huang-29.

Pan et al. (2010) designed and synthesized a series of new 2-arylimino-3-aryl-thiazolidine-4-ones compounds based on the thiazolidione core structure of Qin-2 to acquire more effective and less toxic HK inhibitors, and six derivatives (Pan-10, Pan-12, Pan-20, Pan-27, Pan-28, and Huang-29, Figure 5 ) were synthesized by modifying the functional groups through cyclisation, aldol condensation, substitution, and hydrolysis reactions Huang et al. (2012). All six derivatives showed concentration-dependent inhibition of the autophosphorylation activity of WalK, with IC_50_s of 88.35, 61.15, 34.83, 66.68, 22.15, and 82.51 μM, respectively, which are comparable to Qin-2 (IC_50_ = 47.9 μM).

The bactericidal and biofilm-killing activities of Pan’s derivatives were significantly improved by comparing to Qin-2. It was observed that Pan-12, -20, -28, -27, and Huang-29 showed significant antibacterial activities against *S. epidermidis* and *S. aureus* with MICs ranging from 1.5 μg/ml to 6.6 μg/ml, and the antibacterial activity of Pan-10 was lower with MICs of 12.2 and 24.3 μg/ml against *S. epidermidis* and *S. aureus* respectively. All derivatives showed strong bactericidal activities against *S. epidermidis* ATCC35984 both in immature (6 h-old) biofilms and mature (24 h-old) biofilms, and eliminated proliferation of the immature biofilm. None of the derivatives displayed significant effects on the growth of *E. coli* strain ATCC 25922, cytotoxicity on Vero cells, or hemolysis induction in human erythrocytes (Huang et al., 2012).

**FIGURE 6:**
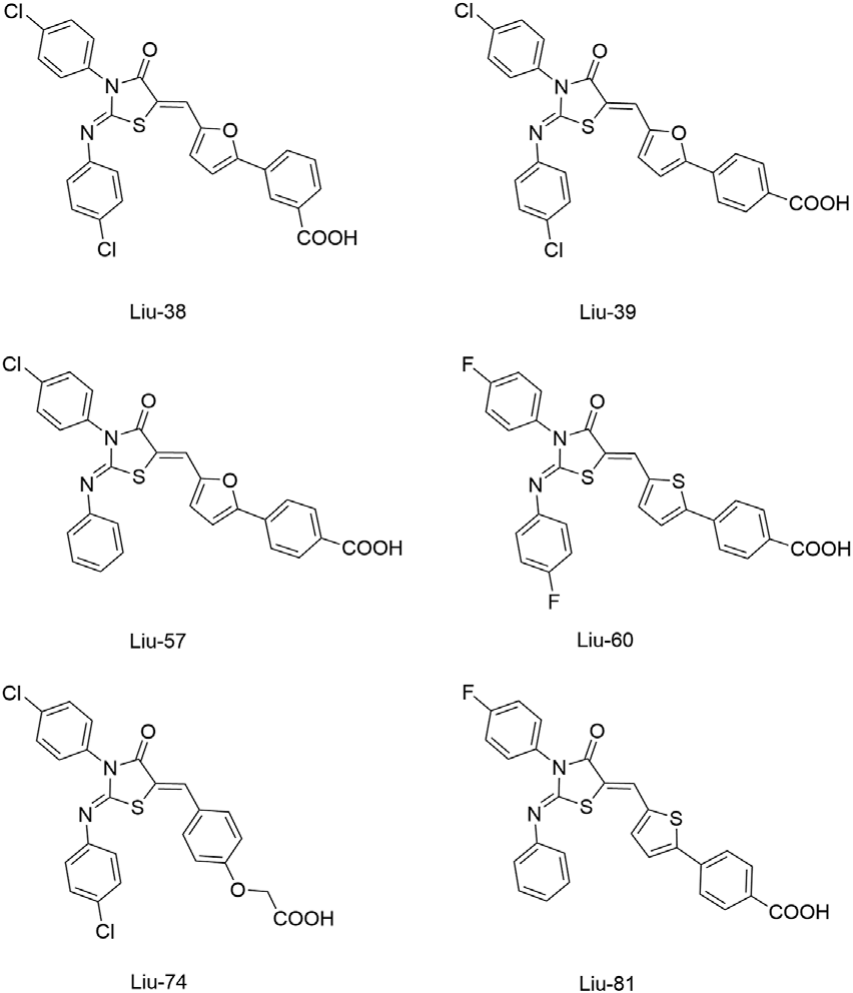
The chemical structures of Liu-38, Liu-39, Liu-57, Liu-60, Liu-74, and Liu-81.

To improve the antimicrobial activity and reduce the toxicity of Qin-2, another six analogs (Liu-38, Liu-39, Liu-57, Liu-60, Liu-74, and Liu-81, Figure 6) of Qin-2 were designed and synthesized by modifying functional groups. They showed inhibitory activities on the autophosphorylation of WalK, with IC_50_ values ranging from 24.2 to 71.2 μM. These compounds displayed good antibacterial activities against *S. epidermidis* and *S. aureus* (MICs of 1.5–6.3 μM), including clinical methicillin-resistant *S. epidermis* (MRSE) and MRSA, which were significantly improved by comparing to Qin-2. All derivatives displayed antibiofilm activities against both the immature and mature biofilms of *S. epidermidis* ATCC 35984, and no significant cytotoxicity or hemolytic activities were observed at the concentration of 200 mM, indicating higher safety of these derivatives than the prototype compound Qin-2. Liu-74, and Liu-81 were further evaluated *in vivo* in the rabbit subcutaneous biofilm infection model, and the reduced bacterial viabilities were observed, indicating the potential efficacies against clinical biofilm infections *in vivo* (Liu et al., 2014).

By introducing chlorine and fluorine substitutions, Zhao et al. (2014) *.* designed a series of thiazolo [3, 2-a] pyrimidine-3-one derivatives based on Qin-5 as the leading compound. The majority of these compounds showed significant activities against *S. epidermidis* and *S. aureus*. Among them, compound Zhao-4e, which contains four Cl substituents, exhibited a high-level inhibitory activity against WalK (IC_50_ of 12.65 μM). Additionally, Zhao-4e showed stronger antibacterial activities against *S. epidermidis* and *S. aureus* than cefazolin. The MICs of Zhao-4e against *S. epidermidis* ATCC35984, *S. epidermidis* ATCC12228, and *S. aureus* ATCC25923 were 1.143, 2.289, and 4.578 μg/ml, respectively. Same as the other thiazolidione derivatives, Zhao-4e didn’t show antibacterial activity against gram-negative strains including *E. coli* and *S. flexneri*. Zhao-4e presented a promising antibiofilm activity against *S. epidermidis* ATCC 35984 at 36.6 μg/ml and showed no hemolytic activity on human erythrocytes at the same concentration.

**FIGURE 7:**
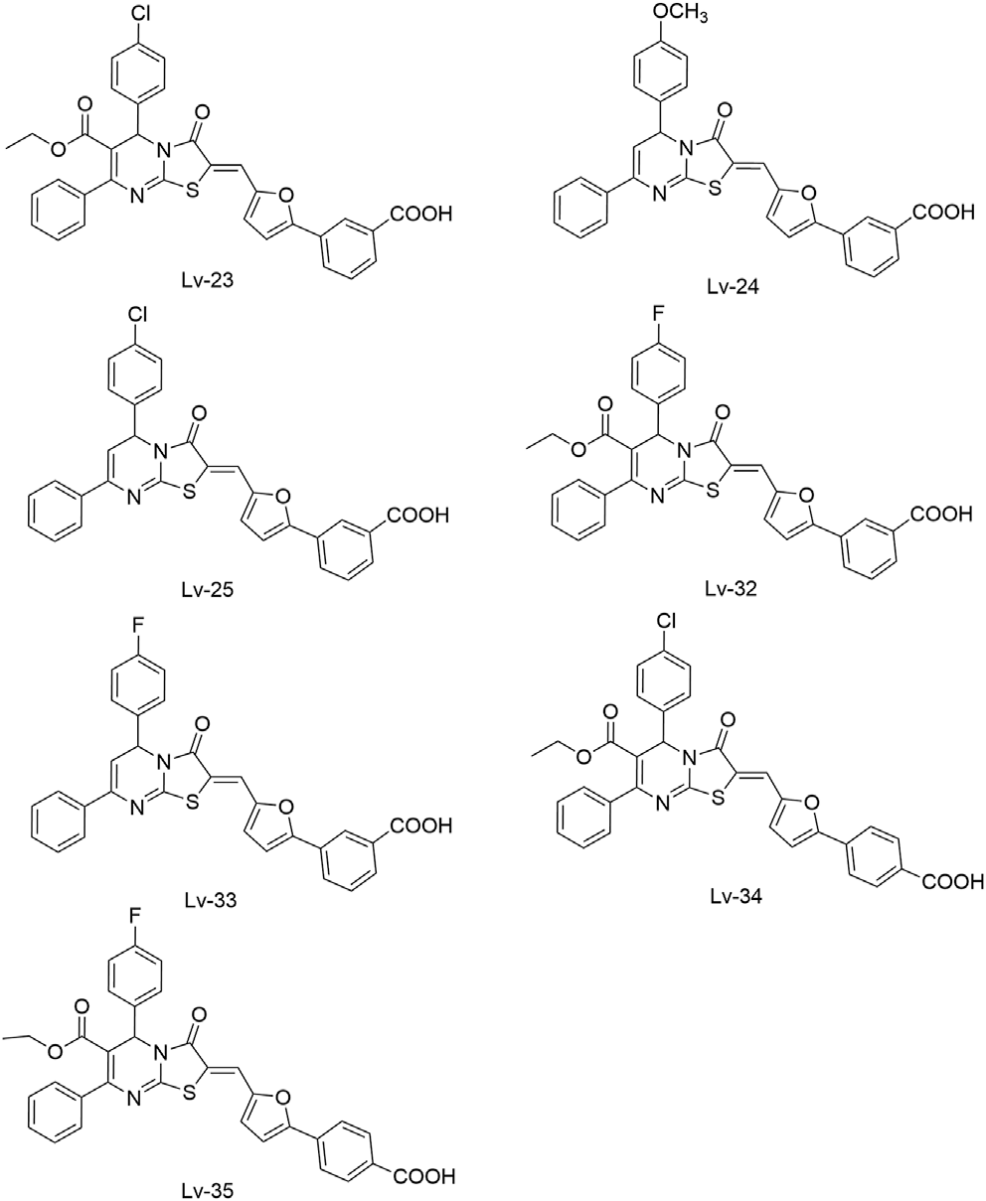
The chemical structures of Lv-23, Lv-24, Lv-25, Lv-32, Lv-33, Lv-34, and Lv-35.

Lv et al. (2017) reported another series of Qin-5’s analogs, represented by seven derivatives (Lv-23, Lv-24, Lv-25, Lv-32, Lv-33, Lv-34, and Lv-35, Figure 7). They exhibited concentration-dependent inhibitions on the autophosphorylation activity of WalK, with IC_50_ values ranging from 61.94 to 83.91 µM.

All seven derivatives displayed higher antibacterial and antibiofilm activities against *S. epidermidis* and *S. aureus* (with MICs ranging from 1.56 to 6.25 µM) than that of Qin-5, and they showed bactericidal activities with the minimum bactericidal concentration (MBC, defined as the minimum concentration of an antimicrobial drug that is bactericidal.) values ranging from 12.5 to 100 µM (4× to 32×MIC), respectively. Lv-32, Lv-33, Lv-34, and Lv-35 exhibited a rapidly bactericidal activity against *S. epidermidis* with a 3-log reduction (CFU/ml) within 1 h, while vancomycin only caused a decrease of about 1 log after 1 h. All the derivatives reduced the proportion of viable cells in mature biofilms and showed no cytotoxicity on Vero cells or obvious hemolysis to human erythrocytes. However, Lv-34 and Lv-35 performed limited aqueous solubilities, which may hinder their further development as potential drug candidates.

In summary, the newly designed thiazolidione derivatives we mentioned above get some improvements on their antibacterial activities, but further structural modifications are still needed to improve the drug-like properties, and increase their antibiofilm and antibacterial properties for the treatment of biofilm-associated infections and multidrug-resistant bacterial infections.

#### 2.1.4 Thiophene

**FIGURE 8:**
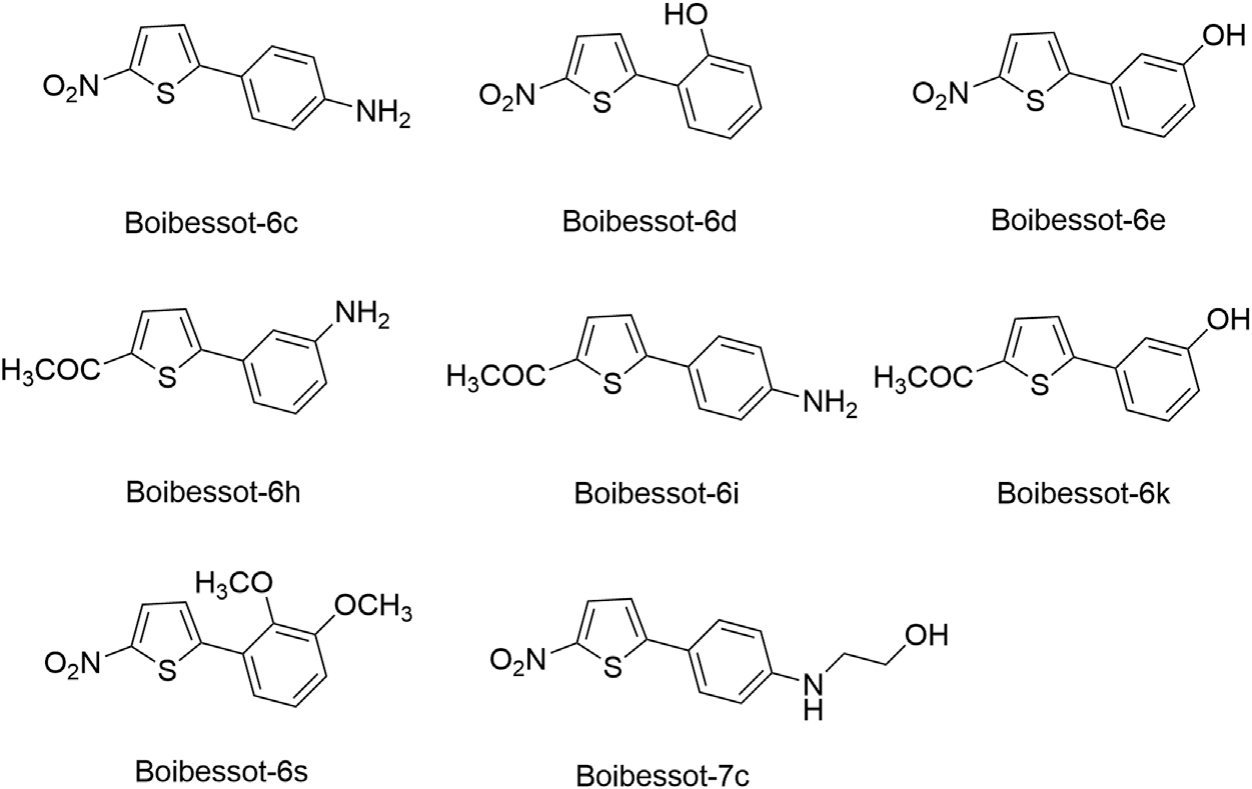
The chemical structures of thiophene derivatives.

Based on the crystal structure of the ATP pocket of WalK, by using structure-based drug design, a series of thiophene derivatives were synthesized through the palladium-catalyzed Suzuki-Miyaura cross-coupling reaction (Boibessot et al., 2016). Among these inhibitors, eight compounds (Boibessot-6c, -6d, -6e, -6h, -6i, -6k, -6s, and -7c, Figure 8) were discovered to inhibit the autophosphorylation activity of the HKs WalK, PhoR, and ResE from *B. subtilis in vitro*, with IC_50_ ranges of 52.81–196.9, 1.63–122.6, and 20.3–243.9 μM, respectively. Moreover, these compounds did not inhibit the DNA-gyrase in *E. coli*, which contains a structurally related CA domain with HKs (Boibessot et al., 2016).

Among these eight compounds, Boibessot-6d and -6e exhibited broad-spectrum antimicrobial activities against both gram-positive and gram-negative bacteria including some important human pathogens, with MICs ranging from 7 μg/ml to 32 μg/ml, while Boibessot-6d was slightly more potent than Boibessot-6e. The MIC of both compounds against *B. subtilis* and *Bacillus anthracis* was 7 μg/ml, which was lower than that against *S. aureus*, *S. pyogenes*, *Listeria monocytogenes*, *S. enterica*, and *E. coli* (ranging from 10 μg/ml to 32 μg/ml). However, Boibessot-6d and -6e showed moderate or poor antimicrobial activities against *E. faecalis*, with MICs of 32 and >64 μg/ml, respectively. Researchers observed that Boibessot-6d could induce slow bacterial lysis over a time course of 9 h which is similar to vancomycin, but Boibessot-6e induced a more rapid cell lysis process, which further indicated that Boibessot-6d and -6e exhibit bactericidal activity rather than bacteriostatic. Additionally, Boibessot-6d could be used as an adjuvant at the concentration of 25 μg/ml combined with amoxicillin or cefotaxime for all the tested *E. faecalis* and *S. aureus* isolates and restored the sensitivity of antibiotic-resistant isolates. Unspecific lytic activity was not observed because both compounds did not exhibit hemolytic activities on sheep red blood cells (Boibessot et al., 2016). Compounds of this series can be considered as potent leads for the development of broadly effective antibacterial agents.

#### 2.1.5 Diaryl Pyrazole

The ATP-binding domain of HK is highly conserved with the ATPase domain of eukaryotic Hsp90 molecular chaperones, suggesting that Hsp90 inhibitors may also function as HKs inhibitors. Vo et al. (2017) screened the ATP-competitive Hsp90 inhibitors against CckA, which is essential for virulence in *Caulobacter crescentus*, and the diaryl pyrazole was identified as a promising scaffold for HK inhibitors. A small library of diaryl pyrazole compounds was synthesized, and among them, compound Vo-5b was identified as one of the most potent HK inhibitors. Moreover, the structural activity relationship (SAR) study indicated the 4-chlororescorcinol group was an important pharmacophore for the inhibitor’s potency.

By using an ATPase-coupled enzyme assay with CckA-ΔTM, a construct of CckA spanning amino acids 70–691 containing the whole cytosolic portion, Vo-5b and other diaryl pyrazole derivatives were discovered as ATP competitive inhibitors, indicating that they may directly bind to the ATP active site. Inhibition assays confirmed that Vo-5b showed more potent inhibitory activity against CckA (IC_50_s of 28 μM) than PhoQ (IC_50_s of 328 μM), an HK in *Salmonella* that is essential for virulence, and showed no inhibitory activity against DivJ, an HK in *Caulobacter crescentus* that regulates cellular differentiation (Vo et al., 2017). Vo-5b showed *in vitro* inhibitory effects against *C. crescentus*, *B. subtilis*, and *E. coli* with MICs of 74.4, 49.6–74.4, and 12.4–24.8 μg/ml, respectively (Vo et al., 2017).

#### 2.1.6 Velikova-13

Velikova et al. (2016) reported the identification of putative HK autophosphorylation inhibitors by using a combination of *in silico* and *in vitro* fragment-based screening by differential scanning fluorimetry. Velikova-13 was discovered as the most potent compound, inhibiting the autophosphorylation of HK in a concentration-dependent manner, with IC_50_s against *S. aureus* and *E. coli* HK PhoR of 212 and 16 μM, respectively.

Velikova-13 mainly showed antibacterial activity against gram-positive pathogens and could inhibit the growth of tested *S. aureus,* including methicillin-resistant *S. aureus* (MRSA), *S. epidermidis,* and *S. pneumoniae* (MICs of 8–16, 1–16, and 16 μg/ml, respectively). Velikova-13 was bactericidal for the tested gram-positive strains, including *S. aureus* DSM 20231 (MBC = 33 μg/ml), MRSA (MBC ≤16 μg/ml) and *S. epidermidis* DSM 20044 (MBC = 8 μg/ml), while Velikova-13 showed no effects against the tested gram-negative strains including *K. pneumoniae* and *P. aeruginosa* (MIC > 500 μg/ml) (Velikova et al., 2016). However, the MICs against *S. aureus* and other gram-positive bacteria were significantly lower than the IC_50_s against *S. aureus* PhoR, which indicated that antibacterial activities of Velikova-13 may be caused by other unknown mechanisms.

The result of native polyacrylamide gel electrophoresis suggested that inhibitory activities of Velikova-13 and other compounds are unrelated to the protein aggregation. The molecular docking analysis (without experimental confirmation) predicted that Velikova-13 may bind to the ATP-binding site in the CA domain (Velikova et al., 2016).

#### 2.1.7 Five Traditional Chinese Medicine Monomers Targeting VicK

Targeting the ATPase domain of VicK, by using the structure-based virtual screening on a natural Traditional Chinese Medicine (TCM) monomers library (Li et al., 2009; Zhang et al., 2015), five TCM monomers, Zhang-1, -2, -3, -4, -5, were identified by Zhang et al. (2015), which specifically inhibited the autophosphorylation of VicK in dose-dependent manners, showing IC_50_ values of 3.8, 5.4, 15.4, 4.6, and 9.1 µM, respectively.

The antibacterial results showed that the MICs of Zhang-1, -2, -3, -4, and -5 against *S. pneumoniae* were 37.1, 38.5, 17, 68.5, and 21 μg/ml, respectively, and all of these five compounds showed antimicrobial effects against penicillin-resistant *S. pneumoniae*, especially Zhang-3 and Zhang-5 showed moderate antibacterial activities with 50% minimum inhibitory concentration (MIC_50_, defined as the minimum inhibitory concentration required to inhibit the growth of 50% of organisms.) values of 17 and 42 μg/ml, respectively. Besides, these five compounds also showed antibacterial activities against *S. mutans*, *S. pyogenes*, *S.mitis*, and *S. pseudopneumoniae*. Importantly, Zhang-3 and Zhang-5 also displayed inhibitory activities against MRSA, with MIC of 34–68 and 84.4–336 μg/ml, respectively. Zhang-3 and -5 showed significantly synergic antimicrobial activity with penicillin *in vivo* and *in vitro*, and effectively reduced nasopharyngeal and lung colonization caused by different penicillin-resistant pneumococcal serotypes, and they also performed synergic antimicrobial activities with erythromycin and tetracycline. *In vivo* experiments showed that the survival time of mice infected by *S. pneumoniae* NCTC7466 could be prolonged by the treatment of these five compounds. The time-killing assays showed that the compounds elicited bactericidal effects against *S. pneumoniae* D39, which led to a 6-log reduction in colony-forming units after exposure to compounds at 4 × MICs for 24 h. The crystal violet staining revealed that the five compounds at MIC or sub-MIC could inhibit the formation of *S. pneumoniae* biofilm, without significant toxicity on Vero cells, which reflects a good safety (Zhang et al., 2015). The present study provides evidence of the antibacterial efficacies of the five leading compounds against pneumococcus and other gram-positive bacteria, and they may serve as an alternative strategy for the treatment of bacterial infections.

### 2.2 Inhibitors Targeting the Dimerization and Histidine Phosphorylation Domain

#### 2.2.1 Signermycin B

By screening over 10,000 *Streptomyces* extracts with sensitive differential growth assays, signermycin B was discovered by Watanabe et al. as the first antibiotic targeting at the WalK dimerization domain according to surface plasmon resonance (SPR) experiments (Watanabe et al., 2003; Watanabe et al., 2012).

Signermycin B exhibited the WalK inhibitory activities (including *S. aureus*, *E. faecalis*, *B. subtilis*, and *S. mutans*, with IC_50_s ranging from 37 to 62 μM) and well antimicrobial activities (including MRSA and vancomycin-resistant *E. faecalis*, with MIC values ranging from 3.13 μg/ml to 6.25 μg/ml) against gram-positive bacteria (Watanabe et al., 2012). It can be noticed that the MICs against the tested gram-positive bacteria were significantly lower than the IC_50_s against WalK, which indicated that WalK may not be the only target of signermycin B and there could be some other molecular mechanisms for Signermycin B’s antibacterial activities.

In the WalK expression experiment, the antibacterial activities of signermycin B were significantly decreased against WalK-induced cells, but such phenomenon was not observed in the WalR-overexpression cells, which indicated that signermycin B could specifically target at WalK. Following SPR experiment and competition assay with ATP indicated that signermycin B binds directly to the dimerization domain of WalK of *B. subtilis* and showed no ATP competition effects, which suggested that signermycin B contains a different mechanism, unlike most HK inhibitors targeting the ATP binding site. Protein cross-linking was performed in the presence of the cross-linker glutaraldehyde to explore the mechanism of action of signermycin B, which interfered with the cross-linking of WalK dimers rather than causing the protein aggregation. Moreover, for *B. subtilis* and *S. aureus*, signermycin B preferentially down-regulated the expression of WalR regulon genes including *yocH*, *yvCE*, *yoeB*, *yjeA*, *isaA*, and *ssaA in vivo*, thereby it can inhibit the cell division (Watanabe et al., 2012). The above findings suggested that the dimerization domain of WalK could be considered as a potential binding site in the discovery of novel antibacterial agents, and supported the further development of signermycin B as an antimicrobial against drug-resistant bacteria.

#### 2.2.2 Waldiomycin

Focusing on the discovery of new antibacterial agents, Igarashi et al. (2013) screened metabolites from *Streptomyces* sp. MK844-mF10 to isolate HK inhibitors by using a differential growth assay they previously reported (Okada et al., 2007), and the waldiomycin was isolated as a WalK inhibitor, composing of 1,3-dioxolane-2-carboxylic acid linked to an angucyclic polyketide *via* a tetraene linker and tetrahydropyran, which belongs to angucycline antibiotics and structurally relates to dioxamycin (Sawa et al., 1991).

Waldiomycin showed moderate antibacterial activity against *S. aureus* and *B. subtilis*, including methicillin-resistant strains, with MICs ranging from 8 μg/ml to 16 μg/ml, but showed no activity against *E. faecalis*, *M. smegmatis*, and all the tested gram-negative bacteria (Igarashi et al., 2013). Among the tested strains, the antimicrobial activity of Waldiomycin against *S. aureus* at a comparable level with other angucyclinone antibiotics, such as aquayamycin (MIC = 6.25–12.5 μg/ml) (Sezaki et al., 1968), rabelomycin (MIC = 6.3 μg/ml) (Liu et al., 1970), and sakyomicin A (MIC = 9.38 μg/ml) (Nagasawa et al., 1984).

Waldiomycin can inhibit the autophosphorylation activities of WalK from *B. subtilis*, *S. aureus*, *E. faecalis*, and *S. mutans*, with IC_50_s of 10.2, 8.8, 9.2, and 25.8 µM, respectively (Fakhruzzaman et al., 2015). The qRT-PCR analysis of WalR regulon genes suggested that waldiomycin repressed the WalK/WalR system in *B. subtilis* and *S. aureus* thereby inhibiting the expression of several cell wall metabolism genes. The morphology of *S. aureus* cells treated with waldiomycin displayed an increased aggregation, rather than the proper cellular dissemination. The autolysis experiment showed that the waldiomycin-treated *S. aureus* cells showed high resistance to Triton X-100-induced and lysostaphin-induced lysis, and these cell lysis phenotypes are consistent with those of cells starved for the WalK/WalR. Since the WalK/WalR system can regulate autolysis, these results further indicated that waldiomycin could inhibit WalK. Furthermore, it was confirmed that waldiomycin inhibited the WalK autophosphorylation *in vivo* by observing the phosphorylated WalK ratio in cells using Phos-tag sodium dodecyl sulfate polyacrylamide gel electrophoresis (SDS-PAGE) (Fakhruzzaman et al., 2015). Further research showed that waldiomycin not only inhibited WalK of gram-positive bacteria but also blocked the activities of most class-I HKs in gram-negative bacteria, including *E. coli* QseC, EnvZ, and PhoQ with IC_50_s of 15.1, 22.4, 12.5 µM, which suggested that waldiomycin was a general inhibitor of class-I HKs (Eguchi et al., 2017).

A series of experiments showed that waldiomycin inhibited HK in an ATP-non-competitive manner, suggesting that the binding site of waldiomycin may not be located in the ATP active site in the CA domain. Additionally, more detailed experiments, including nuclear magnetic resonance (NMR) spectroscopy and protein amino acid mutation, indicated that waldiomycin may bind to the H-box region of the DHp domain, which contains the conserved His residue, to inhibit the class-I HKs and suppress the autophosphorylations (Eguchi et al., 2017; Kato et al., 2017). The broad-spectrum HK inhibition activities of waldiomycin showed that the H-box could be a promising target for the design of novel broad-spectrum HK inhibitors especially those HKs with the WalK-type H-box region. Besides, the binding interactions between waldiomycin and *S. aureus* WalK were analysed by Radwan and Mahrous (2020) through molecular docking studies and molecular dynamics simulations, which indicated that the amino acid residue Lys100 was the key residue for hydrogen bonding with waldiomycin at the binding site and possibly important for the enzyme activity. Studies also showed that when the carboxylic acid of waldiomycin was replaced by the methyl ester group, the antibiotic activity disappeared (Radwan and Mahrous, 2020; King and Blackledge, 2021).

### 2.3 Inhibitors Targeting the Histidine Kinase Sensor Domain

#### 2.3.1 Maprotiline

By screening a molecular library containing 420 FDA-approved drugs based on the TCS-dependent biofilm effect, maprotiline, an FDA-approved tetracyclic antidepressant drug, was discovered with the potential to combat *Francisella* infection (Dean and van Hoek, 2015).

Maprotiline effectively reduces the biofilm formation by affecting *Francisella* QseC. Although the exact molecular mechanisms still need to be elucidated, based on computational modeling, researchers predicted the periplasmic sensor domain of QseC might be the binding site of maprotiline (Dean and van Hoek, 2015). Moreover, maprotiline could significantly downregulate the expression of IglC, which is a known *Francisella* virulence factor, in a manner of QseC dependence, as the same decrease in IglC expression was not observed in the qseC mutant. Further *in vivo* studies have demonstrated that maprotiline can prolong the time of disease onset and survival in *F. novicida*-infected mice and waxworm, which indicated that maprotiline may not only disrupt TCS signaling *in vitro* to decrease the formation of biofilm, but also decrease the virulence of *Francisella in vivo* through disrupting the expression of TCS-dependent virulence factor (Dean and van Hoek, 2015).

Maprotiline showed strong antivirulence activity without direct inhibition on the growth of bacteria, which indicated maprotiline was less likely to confer bacterial resistance. Such an antivirulence approach could be a new direction for the drug discovery against antibiotic-resistant pathogens or could be used in conjunction with other known antibiotics to potentiate the antibacterial activities. Further investigations on maprotiline as a potential drug candidate to treat infections caused by *Francisella* and other pathogens would be expected to generate novel anti-bacterial drugs (Dean and van Hoek, 2015; Feldmann and Cavanagh, 2015).

### 2.4 Inhibitor as a Prodrug

#### 2.4.1 LED209

**FIGURE 9:**
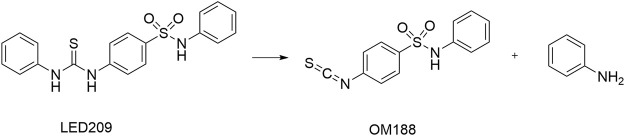
Upon interaction with QseC, LED209 breaks into the active component of OM188 and an aniline group (Curtis et al., 2014).

LED209 was identified by Rasko et al. (2008) from HTS of a library containing 150,000 small organic compounds. It selectively inhibited the binding of the signaling molecules to QseC from different gram-negative pathogenic bacteria. Studies showed that LED209 at very low level of concentrations (e.g., 5 pM *in vitro*) could block the autophosphorylation of QseC triggered by both the host stress hormones (norepinephrine or epinephrine) and the bacterial signal AI-3, and the subsequent virulence gene expression, but did not influence the bacterial growth. The latest research has revealed the unique action mode of LED209 as a prodrug, by which it loses an aniline group and releases a warhead of isothiocyanate OM188 (Figure 9). With this unique mechanism, OM188 can avoid being metabolized and/or degraded. The warhead OM188 allosterically modifies QseC by convalently binding to Lysines (K256 and K427) of the HK, impairs the function of QseC, and prevents the activation of the virulence program of several gram-negative pathogens both *in vitro* and during the treatment of *Salmonella typhimurium* and *Francisella tularensis* murine infection. Besides, LED209 can decrease the formation of biofilm in the enteroaggregative *E. coli* O104:H4 and in several multidrug-resistant clinical isolates of recurrent urinary tract infections. Pharmacokinetics and toxicology studies have shown that LED209 had desirable pharmacokinetics and safety *in vitro* and in rodents. However, the plasma exposures with LED209 were high, which indicated the limited penetration of the blood-brain barrier (Curtis et al., 2014).

Recently, researches focused on the combination or conjugation of LED209 with other antibacterial molecules open an area for broader applications of LED209. Poly (amidoamine) (PAMAM), which was reported as a broad-spectrum antimicrobial agent, has limitations as its cytotoxicity to mammalian cells and poor selectivity. Xue et al. (2015) conjugated G3 PAMAM with LED209 to generate a multifunctional agent PAMAM-LED209 which showed higher selectivity on gram-negative bacteria and lower cytotoxicity to mammalian cells due to the reduced positive charges in G3 PAMAM surface than using PAMAM alone. The conjugate not only remained the strong antibacterial activity, but also inhibited virulence gene expression of gram-negative bacteria without inducing resistance development of the pathogens. A subsequently obtained chitosan (CS)-LED209 was also reported to have a higher selectively antimicrobial and anti-adhesion activity against MDR-*E. coli* as well as the lower cytotoxicity to the mammalian cell than CS (Zhou et al., 2019). An all D-amino acid analogue of Tachyplesin I (TPAD) which belongs to cationic β-hairpin antimicrobial peptides with broad-spectrum antimicrobial activity was reported, but it was found that TPAD tends to induce bacterial resistance by activating the QseC/B TCS. The combination of TPAD with LED209 could significantly enhance the bactericidal effect against MDR gram-negative bacteria such as *Pseudoalteromonas* and *S. maltophilia* , which opens an avenue to improve the antimicrobial efficiency of antimicrobial peptides (Yu et al., 2020). Moreover, Zhou et al. (2021) covalently modified cellulose membrane (CM) with LED209 *via* click reaction to produce an antibacterial material (LED209-CM). LED209-CM exhibited marked anti-adhesion ability to enterohemorrhagic *E. coli*, and significantly reduced the formation of bacterial biofilm. LED209-CM was also able to repress the expression of virulence genes in enterohemorrhagic *E. coli* and indicated no cytotoxicity to mammalian cells. This construction strategy of antibacterial surface expanded the application potential of LED209 in implant-related infections.

### 2.5 Inhibitors With Targets Under Validation

#### 2.5.1 Walkmycins

##### 2.5.1.1 Walkmycin B

Okada et al. screened 1,368 cultures of *Streptomyces* sp. with differential growth assays developed from a temperature-sensitive walR mutant of *B. subtilis*, and walkmycin A, B, and C, which were produced by the strain MK632-100F11, were discovered (Okada et al., 2007; Okada et al., 2010).

Among these three walkmycins, walkmycin B is the major product. SPR showed that the equilibrium dissociation constant (K_D_) was 7.63 μM, indicating that walkmycin B is specifically bound to WalK of *B. subtilis* with a relatively high affinity (Okada et al., 2010). By densitometrically quantifying the autophosphorylation bands, IC_50_s of walkmycin B against WalK of *B. subtilis* and *S. aureus* were found to be 1.6 and 5.7 μM, respectively (Okada et al., 2010). In terms of biological activity, walkmycin B showed potent antibacterial activities against gram-positive bacteria, among which the MICs against *B. subtilis*, *S. aureus*, and *E. faecalis* were ranged from 0.20 μg/ml to 6.25 μg/ml (Okada et al., 2010).

##### 2.5.1.2 Walkmycin C

According to the study by Eguchi et al. (2011), walkmycin C, which can inhibit the autophosphorylation activities of three HKs (VicK, CiaH, and LiaS) of *S. mutans in vitro*, repressed at least two virulence factors and blocked the genetic competence of *S. mutans in vivo*. Walkmycin C showed antimicrobial activities against all tested gram-positive bacteria, including *S. aureus*, *B. subtilis*, *E. faecalis*, *S. pneumoniae*, and *S. pyogenes*, with MICs ranging from 0.0625 μg/ml to 16 μg/ml, without showing antimicrobial activities against the tested gram-negative bacteria (Eguchi et al., 2011).

In addition to the WalK HKs in *B. subtilis* and *S. aureus*, walkmycin C showed IC_50_s of 2.53, 4.29, and 4.96 μg/ml (2.87, 4.87, and 5.63 μM) against the autophosphorylation activities of the cytoplasmic domains of VicK, CiaH, and LiaS, respectively, from *S. mutans*. Moreover, it also inhibited the autophosphorylation activities of EnvZ and PhoQ from *E. coli*, both with IC_50_s of 1.25 μM. Studies of the inhibitory activity of walkmycin C against the virulence factors of *S. mutans* showed that the exposure of walkmycin C at sub-MICs level could inhibit the biofilm formation, acid tolerance, and competence. The experiments about biofilm formation, cell morphology, etc. after treatment of four HK mutants (*vicK*, *liaS*, *ciaH*, and *comD*) with walkmycin C indicated that walkmycin C can repress the activity of at least HKs VicK and CiaH of *S. mutans* (Eguchi et al., 2011).

#### 2.5.2 Two Traditional Chinese Medicine Monomers Targeting AgrC

Zhang et al. (2019) constructed an artificial proteoliposome-based system containing an artificial cell membrane with full-length AgrC, which contains a high similarity with the actual cellular environment. The model was used to screen inhibitors for AgrC among a molecular library containing 14 traditional Chinese medicine monomers by incorporating AgrC into liposomes *via* a detergent-mediated method. Among these monomers, two TCM monomers, rhein, and aloeemodin were screened out. Rhein and aloeemodin can inhibit the autophosphorylation of AgrC, with IC_50_ values of 13.7 and 62.2 μM, respectively. Rhein and aloeemodin showed inhibitory activities on the growth of *S. aureus* in a concentration-dependent manner, with MICs of 32 and 64 μg/ml, respectively. Biofilm formation decreased significantly approximately 20.0% and 33.3% respectively when *S. aureus* was treated with rhein and aloeemodin at the concentrations of 1/2 × MICs. Subinhibitory concentrations of these compounds can significantly reduce the expression of three virulence factors (*hla*, *clfA*, and *clpP*) in *S. aureus* which was regulated by the *agr* system. The results preliminarily confirmed these two traditional Chinese medicine monomers can be the targeting inhibitors of AgrC.

#### 2.5.3 Four Potential PhoQ Inhibitors

The PhoP/PhoQ system modulates the *Shigella flexneri* virulence and stress responses of Mg^2+^, pH, and antibacterial peptides (Lin et al., 2017). Using HTS and enzymatic activity-coupled assays, Cai et al. (2011) reported four compounds Cai-1, -2, -3, and -4 as potential PhoQ inhibitors.

These four compounds inhibited the autophosphorylation activity of *S. flexneri* PhoQc and displayed high binding affinities to the *S. flexneri* PhoQc protein in the SPR response (K_D_ = 4.50, 10.6, 7.56, and 9.40 μM, respectively). Luminescent kinase assays showed that the IC_50_ values of Cai-1, -2, -3, and -4 were 69.37, 48.9, 7.99, and 27.2 μM, respectively. Additionally, the growth curve of *S. flexneri* 9380 showed that the four potential PhoQ inhibitors had no obvious effect on the growth of *S. flexneri* 9380 at 200 μM *in vitro* because the PhoQ/PhoP signaling system did not regulate the cell growth directly. Cai-1, -2, and -3 inhibited the activity of *S. flexneri* 9380 to invade HeLa cells. Mice inoculated with *S. flexneri* 9380 pretreated with four compounds displayed no inflammation, whereas mice inoculated with *S. flexneri* 9380 alone displayed a severe keratoconjunctival inflammation. At the effective concentrations, four inhibitors exhibited the low cytotoxicity and hemolysis of mammalian cells. Thus, these studies demonstrated that all four potential PhoQ inhibitors could reduce the virulence of *Shigella*. The compound structures need to be further modified to increase the efficacy and the stage of infection which is inhibited by these inhibitor candidates needs to be identified (Cai et al., 2011).

#### 2.5.4 Diarylthiazole Derivatives

The PrrBA TCS, one of the four conserved TCSs presented in all mycobacterial species, is critical for the viability of *M. tuberculosis* and is necessary for the initial stage of *M. tuberculosis* infection in macrophages (Tyagi and Sharma, 2003; Haydel et al., 2012). Bellale et al. (2014) screened a representative set (100,000) of the AstraZeneca’s molecular library against *M. tuberculosis* H37Rv, and the MIC, MBC, selectivity index (the ratio of IC_50_ against human A549 cell line to MIC against *M. tuberculosis*), and confirmation of activity by resynthesis were evaluated.

A group of diarylthiazole compounds was identified that contained potent antimycobacterial activities against *M. tuberculosis*. Subsequently, over 40 diarylthiazole derivatives were synthesized, and most of them showed desirable physicochemical properties and potent MICs against *M. tuberculosis* (MIC ≤ 1 μg/ml), such as Bellale-31 and -48, which exhibited significant antimycobacterial activities (*M. tuberculosis* MIC = 0.4 and 0.25 μg/ml, MBC = 0.8 and 0.5 μg/ml, respectively). Moreover, it was observed in the whole genome sequencing of the representative resistant mutant strains that point mutations in *prrb* gene imparted the resistance to the tested diarylthiazole compounds, suggesting that the sensor kinase PrrB may be a target of diarylthiazole (Bellale et al., 2014), but the specific mechanisms of diarylthiazole still need to be clarified.

#### 2.5.5 Xanthoangelol B and the Derivative PM-56

In *S. aureus*, SaeRS is composed of the HK SaeS, RR SaeR, and two auxiliary proteins SaeP and SaeQ. The TCS is regarded as a master virulence regulator because it controls the production of over 20 virulence factors, such as hemolysins, leukocidins, superantigens, surface proteins, and proteases (Liu et al., 2016; Mizar et al., 2018).

Xanthoangelol B was screened out by Mizar et al. (2018) using a GFP (Green fluorescent protein) reporter system they developed previously to screen plant-derived SaeRS inhibitors (Yeo et al., 2018). The compound has been synthesized at present, and a derivative named PM-56 was obtained during the synthesis.

Xanthoangelol B and PM-56 showed excellent inhibitory activities against SaeRS, with IC_50_s against the SaeRS GFP reporter at 2.1 and 4.3 μM, respectively. Significant transcriptional suppression of four downstream virulence genes [α-hemolysin (*hla*), aureolysin (*aur*), γ-hemolysin, and staphylokinase] were observed, which further implied the action of both compounds on the SaeRS pathway and their possibility of development as antivirulence agents (Mizar et al., 2018).

*In vitro*, neither compound suppressed the growth of *S. aureus* at IC_90_ (against SaeRS TCS), especially PM-56, which displayed a low effect on bacterial growth and is therefore considered a desirable antivirulence agent. In an infection model of *G. mellonella* larvae, both xanthoangelol B and PM-56 provided excellent protection to the insects from lethal *S. aureus* infection and reduced bacterial burdens in a dose-dependent manner (Mizar et al., 2018).

The direct binding of xanthoangelol B and PM-56 to SaeS was determined using an intrinsic fluorescence quenching assay, by which the Tyr fluorescence of SaeS at 306 nm was monitored before and after binding of the compounds and the Kd values were calculated according to the percent fluorescence quenching upon ligand binding, and the Kd values of 40 and 6.8 μM for the two compounds to the HK were identified, respectively. Based on the measurements from SDS-PAGE autoradiography and the effects on phosphotransferase (PT) activity of SaeS, both compounds displayed a specific inhibition on HK activity of SaeS, with IC_50_ values of 220 and 160 μM for xanthoangelol B and PM-56, respectively. In additon, Xanthoangelol B and PM-56 could also inhibit another HK, AgrC, with IC_50_ of 339 and 140 μM, respectively, indicating the possibility that xanthoangelol B scaffold could be further developed as a broad-spectrum HK inhibitor despite the IC_50_ values higher than expected (Mizar et al., 2018).

#### 2.5.6 Two Potential DosS and DosT Inhibitors

DosS-DosT/DosR(DosRST) is composed of two heme-based histidine sensor kinases, DosS, and DosT, and a response regulator DosR (Roberts et al., 2004). Zheng et al. (2017) used a DosRST-dependent fluorescent *M. tuberculosis* reporter strain to identify new DosRST inhibitors by using a whole-cell phenotypic HTS to screen a small-molecule library containing ∼540,000 compounds.

Two compounds, Zheng-103A and Zheng-102A, were identified and contained inhibition abilities on the triacylglycerol synthesis, survival, and isoniazid tolerance under the condition of hypoxia, which is associated with the physiological processes of *M. tuberculosis* persistence. Recombinant DosS and DosT proteins were used to conduct biochemical studies to determine the mechanism of action of Zheng-102A and Zheng-103A. Zheng-103A can inhibit the autophosphorylation activity of both DosS and DosT with IC_50_s of 0.5 and 5 μM, respectively, while Zheng-102A only inhibited the autophosphorylation of DosS with IC_50_ of 1.9 μM (Zheng et al., 2017). Their research confirmed that only DosR-controlled pathways are targeted by Zheng-102A, while 13 genes were downregulated in the Δ*dosR* mutant treated with Zheng-103A, which indicated that although Zheng-103A is highly specific for the DosRST pathway, other targets exist (Zheng et al., 2017). Besides, the synergistic interactions of inhibitors showed that the artemisinin, a compound targeting at the sensing domain of DosST heme, was synergistic with Zheng-102A and Zheng-103A, which indicated that the inhibitors’ function by distinct mechanisms could be combined to improve the potency (Zheng et al., 2020). Further studies will be required to evaluate the antibacterial activities and clinical significance of Zheng-103A and Zheng-102A in curing *M. tuberculosis* infection.

## 3 Conclusion and Discussions

In the current review, we summarized more than twenty compounds, and it can be noticed that even aiming at the same binding site, the inhibitors share a great structural diversity, and no obvious structure-activity relationship can be summarized. Besides, it has to be admitted that, at least from our personal views, the current inhibitors discovery studies are still in an early stage, and few promising leading compounds have been obtained yet. SBVS-based compound libraries containing more diverse structures should be constructed for discovering more potential HK inhibitors (Payne et al., 2007). Currently, there is no co-crystal structures have been solved to indicate the interactions between the protein and ligands at the binding sites, which caused that the further structural optimizations could not be effectively directed. In the future, more crystallography and molecular modeling studies should be performed to provide such detailed information. Moreover, most identified inhibitors have highly hydrophobic structures, such as heterocyclic or aromatic rings structures which may influence their druggability (Hirakawa et al., 2020), and it is necessary to optimize the physical and chemical properties of compounds.

Several strategies have been applied to improve the discovery of HK inhibitors and drug design. The combination of HTS with computer aided drug design (CADD) methods, especially SBVS and fragment-based screening (FBS), has been widely applied in drug discovery. By combining with the screening for antibacterial activity, the hits could be identified, and then based on the 3D structures of the target proteins (either obtained by the crystallization or by homology modeling), the hits could be further optimized for better biological activities. Furthermore, given that HKs are transmembrane sensors, recently developed methods for producing active forms of full-length detergent-, liposome-, and nanodisc-solubilize membrane proteins have been used to study and reveal some commonalities and uniquenesses in the structure or function of HKs, which will further help identify broad-spectrum or selective inhibitors, or determine the mechanism of action of some inhibitors (Ma and Phillips-Jones, 2021). These strategies could contribute to the discovery of novel HK-targeted antibacterial agents in the future.

By overviewing the discovery of current HK inhibitors, it can be noticed that different approaches were applied during the identify the target compounds. Several compounds [including Zhang-1, -2, -3, -4, -5 (Zhang et al., 2015), rhein, and aloeemodin (Zhang et al., 2019)] were discovered from the traditional Chinese medicine monomer library, compounds [such as walkmycins (Okada et al., 2010) and signermycin B (Watanabe et al., 2012)] were obtained from the natural antibiotics, and Xanthoangelol B and the derivative PM-56 (Mizar et al., 2018) were discovered from natural products, which indicated that the natural resource is still a major source to identify potential HK inhibitors. The computer aided molecular design studies were widely applied during the discovery HK inhibitors, by combing with various methods, including HTS methods [Benzothiazole (Wilke et al., 2015)], fragment-based screening [Velikova-13 (Velikova et al., 2016)], and structure-based screening [Thiazolidione (Qin et al., 2006)], which exhibited that the computer modelling can play important role in the drug discovery, and more work in this field, including molecular dynamic simulations or QM/MM (Hybrid quantum mechanics/molecular mechanics) simulations, should be performed to increase our understanding of the ligand-protein interactions, especially when the co-crystal structures are unavailable. Besides, the development of *in vitro* biological screening models significantly accelerated the screening process, for example maprotiline (Dean and van Hoek, 2015) discovered based on the TCS-dependent biofilm effects, and walkmycins (Okada et al., 2010) obtained from *Streptomyces* sp. with a differential growth assay.

It can be noticed that although there are a number of hits discovered, the following lead optimization needs to be further advanced. Currently, the HK inhibitors optimizations are mainly targeting at the CA kinase domain by competing with ATP. This strategy allows us to gain experience from the research and development of human kinase inhibitors and apply it to the antibacterial research, which could lead the optimization direction to achieve promising leading compounds. However, we must realize that the selectivity of kinase inhibitors is always a very important issue, so that the differences of kinase domains between bacterial and human must be carefully considered during the structural optimization. In additional to the kinase domain, other potential active sites also deserve continuous attention, and the successful development of such leading compounds will hopefully avoid the selectivity problem of kinase inhibitors, and provide new directions for the drug discovery.

It is worthwhile to notice that many HKs responsible for bacterial virulence, biofilm formation, and antibiotic resistance under stress conditions are non-essential for bacterial survival and growth. Those molecules inhibiting such HKs usually display little or no classical antibacterial effect, which means that routinely used, simple, economical, and easily reproducible *in vitro* assays such as MIC determination are not suitable for the confirmation of the inhibitors’ activities. Then, how to comprehensively and effectively evaluate the varied activities of the compounds *in vitro* and *in vivo* is a subject worthy of continuous attention.

The processes regulated by bacterial HK are so diverse that their inhibitors can be developed as potential bacteriostatic/bactericidal agents, antibiotic synergists, biofilm inhibitors, or virulence inhibitors depending on the physiological functions of different HKs in bacteria. Inhibition of essential bacterial HKs has always been a particularly attractive strategy due to their conserved active sites of the catalytic domains across bacterial strains or species and growth dependence. This also implies that an inhibitor of one HK may in fact block multiple TCS regulatory networks. Compounds that inhibit these HKs offer a great benefit because their targets and mechanisms are definitely different from those of currently used antibiotics, and are not affected by the cross-resistance developed in known MDR pathogens. However, just like “every coin has two sides”, the high structural similarity between bacterial HKs provides advantages to generate inhibitors with well polypharmacological activities and bactericidal effects, but may also tend to allow these inhibitors to act as conventional broad-spectrum antibiotics and drive the evolution of resistance to these molecules (King and Blackledge, 2021). In contrast, those inhibitors that target non-essential bacterial HKs often causes multiple simultaneous but non-lethal phenotypic effects including reduced fitness, impaired biofilm formation, attenuated virulence gene expression, and increased susceptibility to antibiotics, are thought to be less likely to induce resistance due to their relatively mild selective pressure on bacterial growth, thus may represent a particularly exciting area of future research (Curtis et al., 2014). Some laboratory evidence has also shown that inhibiting essential targets would lead to fast(er) development of resistance than inhibition of virulence mechanisms because there would only be selected for resistance in pathogens causing an infection in the host (Mohedano et al., 2005; Velikova et al., 2013). In addition, it can be predicted that bacteria resistant to molecules acting on certain non-essential HKs may be hard to infect the host or be easily eliminated by the host immune system due to loss of virulence or reduced fitness. Taking LED209 that targets QseC as an example, the QseC mutants of *S. typhimurium* and *F. tularensis* generated by the researchers in the laboratory was refractory to treatment with LED209 but also lost their ability to infect mice (Curtis et al., 2014). Certainly, the question of whether and how bacteria from the clinic develop possible resistance to the known HK inhibitors still remains and further studies are needed before a real antimicrobial drug targeting HKs is successfully developed. Overall, increased efforts and resources will be required. Improving our understanding of the interactions between the targeted domain and potential ligands is necessary. Further studies should evaluate how to improve the target specificity and antibacterial activities of the compounds. Further evaluation of antibacterial activity *in vivo*, toxicity trials, and preclinical studies of the compounds, will also be needed before application of the compounds in the clinical setting to treat bacterial infections. Furthermore, it would be interesting to see whether bacteria can spontaneously develop resistance against molecules that inhibit bacterial HKs. In the future, researchers can conduct more prospective analysis at the laboratory level by inducing spontaneously resistant mutants to HK inhibitors that are under development to further demonstrate the target specificity of the molecules and elucidate the possible resistant mechanism of bacteria to them. Although challenging, we believe this strategy of developing HK inhibitors is a viable approach to overcome severe drug resistance.

## Abbreviations

TCS, two-component system; HK, histidine kinase; RR, response regulator; P-RR, phosphorylated response regulator; QS, quorum sensing; SPR, surface plasmon resonance; MRSA, methicillin-resistant *Staphylococcus aureus*; MIC, minimum inhibitory concentration.
